# Cytotoxic, Drug-Interaction, and Apoptosis-Associated Effects of β-Boswellic Acid and Doxorubicin in Murine 4T1 TNBC-like Cells

**DOI:** 10.3390/biomedicines14091978

**Published:** 2026-09-02

**Authors:** Zahide Küçük, Mehmet Cudi Tuncer, Şamil Öztürk

**Affiliations:** 1 Department of Gynecology and Obstetrics, Dr. Zahide Küçük Practice, Ankara 06530, Turkey; drzahidekucuk@outlook.com; 2 Department of Anatomy, Faculty of Medicine, Dicle University, Diyarbakır 21280, Turkey; 3 Vocational School of Health Services, Çanakkale Onsekiz Mart University, Çanakkale 17100, Turkey; ozturksamil@outlook.com

**Keywords:** boswellic acid, doxorubicin, triple-negative breast cancer, apoptosis, mitochondrial membrane potential, *Bax*/*Bcl2*, *Casp3*

## Abstract

**Background/Objectives:** Triple-negative breast cancer (TNBC) is an aggressive breast cancer subtype with limited targeted therapeutic options. Although the anticancer and pro-apoptotic properties of boswellic acids have previously been reported, the interaction profile of chemically defined β-boswellic acid (BA) with doxorubicin (DOX) remains insufficiently characterised in the murine 4T1 TNBC-like model. This study quantitatively evaluated BA–DOX drug interactions using different reference models and characterised the cytotoxic and apoptosis-associated cellular phenotype accompanying combined exposure. **Methods:** Cytotoxicity was assessed using the MTT assay and BA–DOX interactions were evaluated using the Chou–Talalay combination index (CI), highest single-agent (HSA) and Bliss independence models. Apoptosis and cell-cycle distribution were analysed by flow cytometry and mitochondrial membrane potential was assessed by JC-1 staining. Caspase-3/7 activity, RT-qPCR, live/dead Calcein-AM/PI staining, 4′,6-Diamidino-2-phenylindole (DAPI) nuclear staining, and cytokine measurements were also performed. Gene Ontology (GO), Kyoto Encyclopaedia of Genes and Genomes (KEGG), and STRING-based protein–protein interaction (PPI) analyses were used to explore putative molecular pathways associated with experimental findings. **Results:** The 48 h selectivity index of BA was 1.28, indicating only modest differential cytotoxicity between 4T1 cells and HaCaT keratinocytes under the experimental conditions rather than definitive cancer-cell selectivity. Drug-interaction analyses revealed concentration- and model-dependent effects, with the Chou–Talalay analysis indicating synergism in selected intermediate and higher concentration pairs. Under the selected phenotypic-characterisation condition, BA + DOX produced a greater apoptotic response than either single treatment, accompanied by increased G2/M and Sub-G1 fractions, mitochondrial membrane depolarisation, and increased caspase-3/7 activity. This treatment condition was not included in the drug-interaction analysis and was therefore not interpreted as a pharmacologically validated synergistic combination. RT-qPCR demonstrated increased mRNA expression of *Bax*, *Casp3*, and *Casp9*, decreased mRNA expression of *Bcl2*, and a marked increase in the *Bax*/*Bcl2* mRNA ratio. Calcein-AM/PI and DAPI analyses further demonstrated increased cell death and apoptotic nuclear alterations. The measured concentrations of TNF-α and IL-6 in culture supernatants were lower after BA + DOX treatment, whereas IL-10 remained unchanged; however, these cytokine measurements were not normalised to viable cell number and therefore require cautious interpretation. Exploratory bioinformatic analyses identified predicted associations with apoptosis-, mitochondrial-, and cell-cycle-related processes and pathways; however, these database-derived findings were considered hypothesis-generating and not evidence of BA-dependent target engagement or pathway activation. **Conclusions:** Combined BA and DOX exposure produced greater cytotoxic and apoptosis-associated responses than either single treatment in 4T1 cells, whereas formal drug-interaction classifications varied according to concentration and analytical model. The accompanying changes in mitochondrial membrane potential, caspase-3/7 activity, and apoptosis-related gene expression describe a treatment-associated cellular phenotype but do not identify a direct molecular target of BA or establish a causal molecular mechanism. The findings also do not demonstrate TNBC-specific selectivity. Further studies using additional breast cancer and tissue-matched non-malignant models, together with direct target-engagement and functional pathway-validation approaches, are required.

## 1. Introduction

Breast cancer is the most frequently diagnosed malignancy among women worldwide and remains a leading cause of cancer-related mortality. According to GLOBOCAN 2022, approximately 20 million new cancer cases and 9.7 million cancer-related deaths occurred globally in 2022. Female breast cancer accounted for approximately 2.30 million new cases, making it the most commonly diagnosed cancer among women, and approximately 670,000 deaths, which ranks second among cancer-related causes of death in women after lung cancer [1,2]. TNBC, characterised by the absence of the oestrogen receptor, the progesterone receptor, and the human epidermal growth factor receptor 2, is an aggressive breast cancer subtype associated with a poor prognosis, early metastatic dissemination, and limited targeted therapeutic options [3,4]. Due to its high metastatic potential and immunocompetent background, the murine 4T1 TNBC-like model is widely used for preclinical investigations of TNBC biology and evaluation of therapeutic strategies, although it does not fully recapitulate the biological complexity of human TNBC [3,4].

DOX, an anthracycline chemotherapeutic agent, is widely used in breast cancer treatment due to its potent antiproliferative and pro-apoptotic activity. Its antitumor effects are mediated primarily through DNA intercalation, topoisomerase II inhibition, and generation of reactive oxygen species (ROS), which ultimately contribute to cancer cell death [5]. Despite its clinical efficacy, DOX treatment is limited by intrinsic or acquired chemoresistance and cumulative dose-dependent toxicities, particularly cardiotoxicity [5]. Consequently, considerable interest has focused on combination strategies designed to improve cellular responses to conventional chemotherapeutic agents. Natural bioactive compounds have received particular attention as potential adjuncts to chemotherapy due to their ability to modulate multiple cellular pathways associated with proliferation, survival, and apoptosis [6,7,8,9]. Previous studies have shown that combining DOX with selected phytochemicals can enhance apoptotic responses and modify chemosensitivity in breast cancer cells [6,7,8,9].

Boswellic acid is a naturally occurring pentacyclic triterpenoid derived from the oleogum resin (frankincense) of the *Boswellia* species. Boswellic acids comprise a family of structurally related pentacyclic triterpenoids; in the present study, BA was specifically investigated as described in Materials and Methods. Increasing evidence indicates that boswellic acids exhibit anti-inflammatory and anticancer properties associated with inhibition of cell proliferation, induction of apoptosis, suppression of angiogenesis, and modulation of signalling pathways involved in tumour progression [10,11,12]. Experimental studies involving boswellic acids and related derivatives have implicated signalling molecules such as p53, Akt, and NF-κB in treatment-associated cellular responses [10,11,12,13,14]. These previous findings establish the biological plausibility of investigating BA-containing combinations but do not, by themselves, define the interaction profile of chemically defined BA with DOX in 4T1 cells, demonstrate TNBC-specific activity, or identify a direct molecular target responsible for the combined response.

Apoptosis represents a major mechanism through which anticancer agents eliminate malignant cells. Within the intrinsic apoptotic pathway, the balance between the pro-apoptotic protein BAX and the anti-apoptotic protein BCL-2 is an important determinant of mitochondrial apoptotic signalling [15]. Increased BAX activity promotes mitochondrial outer membrane permeabilization, leading to loss of mitochondrial membrane potential (ΔΨm), cytochrome *c* release, activation of caspase-9, and subsequent activation of executioner caspases-3/7 [16,17]. Accordingly, assessment of mitochondrial membrane potential, caspase-3/7 activity, and apoptosis-related gene expression can provide complementary information regarding the cellular phenotype accompanying treatment. However, these downstream endpoints do not identify a direct molecular target of BA or, in isolation, establish a causal mitochondrial mechanism.

In addition to anticancer activity, comparison with non-malignant cells is important for the preliminary evaluation of differential cellular sensitivity. HaCaT cells are immortalised human keratinocytes widely used as a non-malignant reference model in toxicological and pharmacological studies [18]. Although HaCaT cells are not tissue-matched normal mammary epithelial cells and therefore cannot establish breast cancer-specific selectivity, they can provide a preliminary comparison of treatment-associated cytotoxicity between malignant and non-malignant cellular models.

Against this background, the question addressed in the present study was not whether BA possesses anticancer activity per se, which has already been reported, but how chemically defined BA and DOX interact in the murine 4T1 TNBC-like model and whether the resulting interaction classification is consistent across different analytical frameworks. Drug interactions were therefore evaluated independently using the Chou–Talalay combination index (CI), highest single-agent (HSA), and Bliss independence models. Cellular characterisation of the selected combined-exposure condition included cell-cycle distribution, Annexin V/PI apoptosis, mitochondrial membrane potential assessed by JC-1 staining, caspase-3/7 activity, Calcein-AM/PI live/dead staining, DAPI-based nuclear morphology, levels of the inflammatory cytokines TNF-α, IL-6, and IL-10, and expression of the apoptosis-related genes *Bax*, *Bcl2*, *Casp3*, and *Casp9*. GO, KEGG, and protein–protein interaction analyses were additionally performed as exploratory approaches to identify candidate functional associations rather than experimentally validated molecular mechanisms. We hypothesised that combined BA and DOX exposure would produce a greater cytotoxic and apoptosis-associated response than either treatment alone under the selected experimental conditions. The study was not designed to establish uniform pharmacological synergy, a direct target-level mechanism of BA, or TNBC-specific selectivity.

## 2. Materials and Methods

### 2.1. Preparation of BA and DOX Treatment Solutions

β-Boswellic acid (BA; purity ≥ 98%; CAS No. 631-69-6) and doxorubicin hydrochloride (DOX; CAS No. 25316-40-9) were purchased from Sigma-Aldrich (St. Louis, MO, USA). BA was dissolved in dimethyl sulfoxide (DMSO) to prepare a 100 mM stock solution, whereas DOX was dissolved in sterile distilled water to prepare a 10 mM stock solution. Stock solutions were aliquoted and stored at −20 °C, protected from light, until use. For each experiment, working solutions were freshly prepared by diluting the respective stock solutions in complete culture medium to the required treatment concentrations. The final DMSO concentration did not exceed 0.1% (*v*/*v*) in any treatment condition. To maintain equivalent vehicle exposure across experimental groups, DMSO was added to the control and DOX-only groups to achieve the same final concentration (0.1% *v*/*v*) used in the BA-containing groups.

### 2.2. Cell Models and Culture Conditions

In this study, murine 4T1 mammary carcinoma cells (ATCC^®^ CRL-2539 TM; American Type Culture Collection, Manassas, VA, USA) and non-malignant immortalised human HaCaT keratinocytes (Catalogue No. 300493; Cell Lines Service GmbH, Eppelheim, Germany) were used. Both cell lines were maintained in Dulbecco’s Modified Eagle Medium supplemented with 10% fetal bovine serum (FBS) and 1% penicillin–streptomycin (100 U/mL penicillin and 100 µg/mL streptomycin) at 37 °C in a humidified atmosphere containing 5% CO_2_. Cells were routinely monitored for morphology and subcultured at approximately 80–90% confluence using 0.25% trypsin–EDTA. All experiments were performed using cells between passages 3 and 8 after recovery from the original frozen stocks. Cultures were periodically screened using a PCR-based mycoplasma detection assay and confirmed to be mycoplasma-free before experimental use.

### 2.3. Experimental Design and Treatment Strategy

Following a 24-h pre-incubation period, cells were assigned to vehicle control, BA, DOX, or BA + DOX treatment groups. For dose–response experiments, cells were exposed to BA at 5, 10, 20, 30, 40, or 50 µM or to DOX at 0.1, 0.5, 1, 2.5, 5 or 10 µM for 24 or 48 h. Vehicle-treated control cells received complete culture medium containing 0.1% (*v*/*v*) DMSO.

For drug-interaction analysis, BA and DOX were administered simultaneously in the concentration pairs specified for the combination experiments, and their interaction was subsequently evaluated using the approaches described in Section 2.4. These drug-interaction experiments were analytically distinct from the treatment condition used for subsequent mechanistic assays.

Based on 48-h dose–response profiles and individual half-maximum inhibitory concentration (IC_50_) values, BA (30 µM) and DOX (1.5 µM) were selected as working concentrations for mechanistic experiments. Consequently, cells were assigned to four experimental conditions: vehicle control, BA (30 µM), DOX (1.5 µM), and BA + DOX (30 + 1.5 µM), with BA and DOX administered simultaneously in the combination group for 48 h. The 30 µM BA + 1.5 µM DOX condition was selected for the mechanistic characterisation and was not included in the Chou–Talalay CI analysis; therefore, it was not considered a predefined synergistic concentration pair.

All experiments were conducted as three independent biological experiments (*n* = 3). For MTT-based cell viability measurements, each biological experiment included three technical replicate wells per treatment condition. Unless otherwise specified, subsequent mechanistic analyses—including Annexin V-FITC/PI apoptosis and cell-cycle analyses, JC-1-based assessment of mitochondrial membrane potential, caspase-3/7 activity, RT-qPCR, cytokine measurements, Calcein-AM/PI live/dead staining, and nuclear morphology analysis were performed after 48 h of exposure using the selected treatment conditions: BA (30 µM), DOX (1.5 µM), and BA + DOX (30 + 1.5 µM).

### 2.4. Cell Viability and Drug-Interaction Analysis

Cell viability was assessed using the MTT reduction assay. The cells were seeded in 96-well plates at a density of 1 × 10^4^ cells/well in 100 µL of complete culture medium and allowed to adhere for 24 h. Cells were subsequently exposed to the indicated concentrations of BA, DOX, or BA + DOX for 24 or 48 h. Following treatment, 10 µL of MTT solution (5 mg/mL in PBS) was added to each well, corresponding to a final concentration of MTT of 0.45 mg/mL, and plates were incubated at 37 °C for 4 h. The culture medium was then carefully removed and the resulting formazan crystals were solubilised in 100 µL of DMSO. The plates were gently agitated for 10 min at room temperature to ensure complete dissolution of the formazan crystals. Absorbance was measured at 570 nm using a microplate reader (BioTek Instruments, Winooski, VT, USA), with 630 nm used as the reference wavelength for background correction. Cell viability was calculated relative to the vehicle-treated control and expressed as a percentage of control viability.

For each compound and exposure time, concentration–response curves were fitted to replicate-level, vehicle-normalised viability data by nonlinear least-squares regression using a four-parameter logistic model with a variable Hill slope in GraphPad Prism version 9.0 (GraphPad Software, San Diego, CA, USA). Drug concentrations were log_10_-transformed before fitting. The upper and lower response plateaus and Hill slope were estimated from the data, and the IC_50_ was defined as the concentration corresponding to the midpoint between the fitted upper and lower plateaus. The fitted IC_50_ estimates were reported together with their 95% confidence intervals.

**Figure 1:**
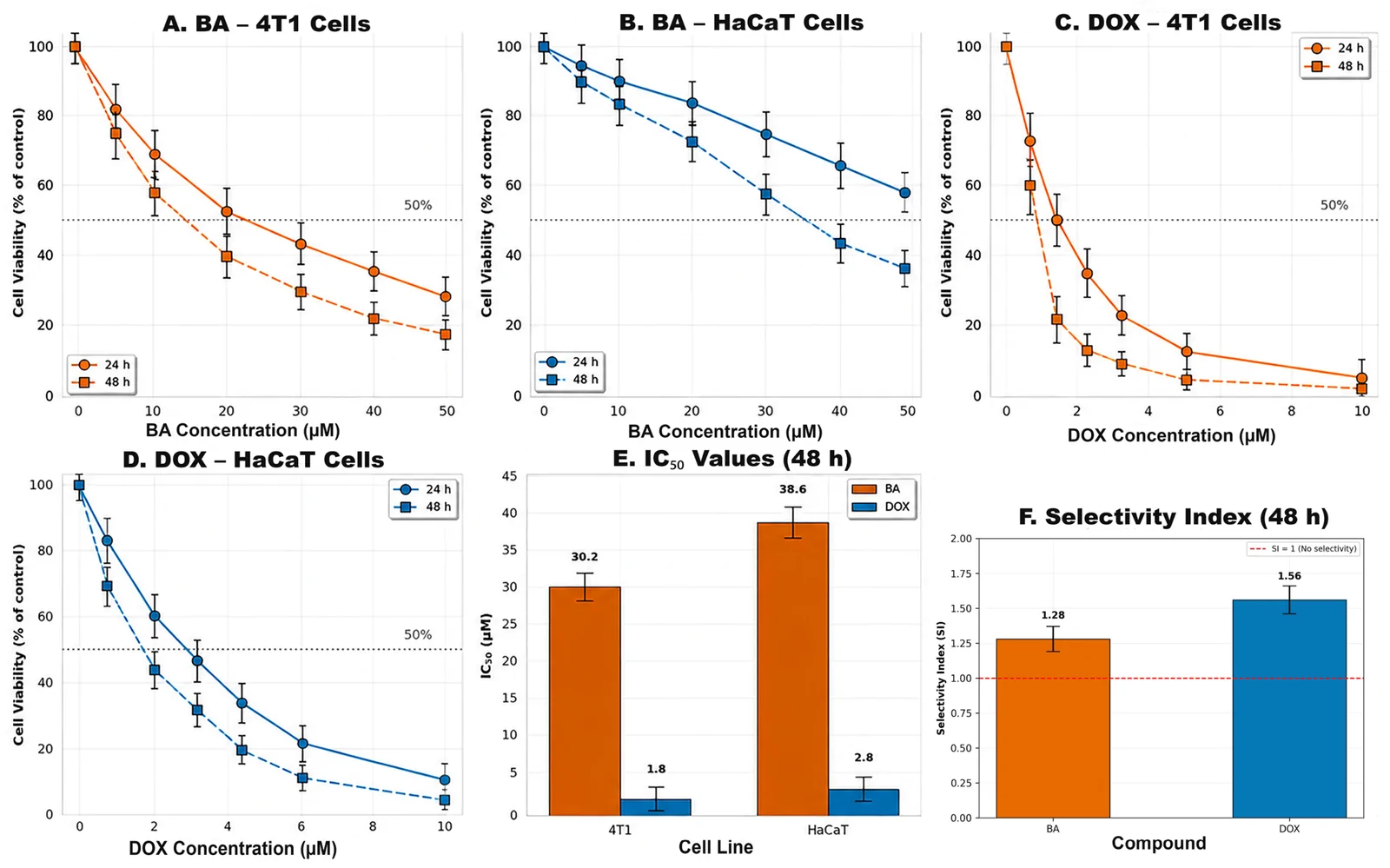
Cytotoxic effects of BA and DOX on 4T1 breast cancer cells and HaCaT keratinocytes. Dose–response curves showing the effects of BA on 4T1 cells (**A**) and HaCaT cells (**B**), and DOX on 4T1 cells (**C**) and HaCaT cells (**D**) after 24 and 48 h of treatment, determined by the MTT assay. Cell viability is expressed as a percentage of the vehicle-treated control. The horizontal dotted line indicates 50% cell viability. (**E**) Comparison of the 48 h IC_50_ values of BA and DOX in 4T1 and HaCaT cells. BA exhibited IC_50_ values of 30.2 μM (95% CI: 28.3–32.1 μM) in 4T1 cells and 38.6 μM (95% CI: 36.4–40.8 μM) in HaCaT cells, whereas the corresponding values for DOX were 1.8 μM (95% CI: 0.6–3.1 μM) and 2.8 μM (95% CI: 1.2–4.4 μM), respectively. (**F**) SI, calculated as IC_50_ (HaCaT)/IC_50_ (4T1) at 48 h. The SI values were 1.28 for BA and 1.56 for DOX, indicating modestly greater cytotoxicity toward 4T1 cells than toward HaCaT cells under the experimental conditions. Data are presented as the mean ± SD of three independent biological experiments (*n* = 3); error bars represent SD.

For drug-interaction analysis, 4T1 cells were simultaneously exposed for 48 h to BA and DOX in a fixed 1:1 concentration ratio using the following concentration pairs: 5 + 5, 10 + 10, 20 + 20, 30 + 30, 40 + 40, and 50 + 50 µM (BA + DOX, respectively). The corresponding single-agent BA and DOX treatments at 5, 10, 20, 30, 40, and 50 µM were evaluated in parallel and used as the reference effects for the HSA and Bliss independence analyses. This exploratory equal-molar concentration series was used to evaluate the interaction profile across a broad range of combined effects; the resulting affected fractions ranged from 0.28 to 0.96. The fixed-ratio interaction experiment was analysed separately from the single-agent concentration–response experiments used to estimate the IC_50_ values presented in Figure 1. The affected fraction (Fa) was calculated as 1 − (cell viability/100). The BA dose, DOX dose, and corresponding Fa value for each concentration pair were entered into CompuSyn software (version 1.0; ComboSyn Inc., Paramus, NJ, USA), and CI values were calculated according to the Chou–Talalay method. For categorical interpretation, an operational tolerance range was applied: CI < 0.8 was classified as synergistic, 0.8 ≤ CI ≤ 1.2 as near-additive, and CI > 1.2 as antagonistic; CI = 1 represented theoretical additivity.

To complement the CI analysis, the fixed-ratio interaction dataset was additionally evaluated using the HSA and Bliss independence models. Treatment effects were expressed as fractional inhibition values ranging from 0 to 1. For the HSA model, the expected effect was defined as the greater effect produced by BA or DOX alone at the corresponding concentration. HSA excess was calculated by subtracting this expected effect from the observed combination effect. For the Bliss model, the expected combined effect was calculated as *E*BA + *E*DOX − (*E*BA × *E*DOX), where *E*BA and *E*DOX represent the fractional effects of BA and DOX alone, respectively. Bliss excess was calculated by subtracting the Bliss-expected effect from the observed combination effect. Excess values were expressed as percentage-point differences. Positive values indicated that the observed combination effect exceeded the corresponding model-expected effect, whereas negative values indicated an effect below the model prediction. Because these interaction models are based on different reference assumptions, the CI, HSA, and Bliss results were evaluated independently rather than requiring concordant classifications between models.

For subsequent phenotypic-characterisation experiments, BA (30 µM) and DOX (1.5 µM) were selected as concentrations close to their fitted 48 h IC_50_ point estimates in 4T1 cells (30.2 and 1.8 µM, respectively), guided by the corresponding dose–response profiles. This treatment pair was analytically distinct from the fixed-ratio concentration series used for CI, HSA, and Bliss analyses and was therefore not classified as synergistic on the basis of these drug-interaction models.

### 2.5. Flow Cytometric Profiling of Apoptosis

Apoptotic cell populations were quantified using the Annexin V-FITC Apoptosis Detection Kit (Catalogue No. V13242; InvitrogenTM, Thermo Fisher Scientific, Waltham, MA, USA). 4T1 cells were treated for 48 h with vehicle control, BA (30 µM), DOX (1.5 µM), or BA + DOX (30 + 1.5 µM), as defined for the mechanistic experiments in Section 2.3. After treatment, cells were harvested by trypsinization, washed twice with cold PBS, and resuspended in the binding buffer supplied with the kit. The cells were then stained with Annexin V-FITC and propidium iodide (PI) according to the manufacturer’s protocol and incubated for 15 min at room temperature in the dark.

The samples were analysed using an FACSCalibur flow cytometer (BD Biosciences, San Jose, CA, USA), with 20,000 events acquired per sample. Flow cytometric data was analysed using FlowJo software (version 10.8.1; BD Life Sciences, Ashland, OR, USA). Cell populations were classified according to Annexin V-FITC/PI staining as viable (Annexin V−/PI−; Q4), early apoptotic (Annexin V+/PI−; Q3), late apoptotic (Annexin V+/PI+; Q2), or necrotic (Annexin V−/PI+; Q1). Total apoptosis was calculated as the sum of the early and late-apoptotic populations (Q3 + Q2).

### 2.6. Flow Cytometric Profiling of Cell-Cycle Distribution

Cell-cycle distribution was evaluated in 4T1 cells after 48 h of exposure to vehicle control, BA (30 µM), DOX (1.5 µM), or BA + DOX (30 + 1.5 µM). Following treatment, cells were harvested, washed with PBS and fixed in ice-cold 70% ethanol at −20 °C for at least 2 h. Fixed cells were subsequently washed with PBS and incubated with RNase A (100 µg/mL) at 37 °C for 30 min to remove cellular RNA. Cells were then stained with PI (PI; 50 µg/mL) for 30 min at room temperature in the dark.

The DNA content was analysed by flow cytometry, with 20,000 events acquired per sample. The resulting DNA-content histograms were analysed using FlowJo software (BD Life Sciences, Ashland, OR, USA), and the proportions of cells in the Sub-G1, G0/G1, S and G2/M phases were quantified.

### 2.7. Quantification of Cytokines in Culture Supernatants

Cytokine concentrations were quantified in culture supernatants collected from 4T1 cells after 48 h of exposure to vehicle control, BA (30 µM), DOX (1.5 µM), or BA + DOX (30 + 1.5 µM). Tumour necrosis factor-alpha (TNF-α), interleukin-6 (IL-6) and interleukin-10 (IL-10) concentrations were measured using commercially available ELISA kits (Thermo Fisher Scientific, Waltham, MA, USA) according to the manufacturer’s instructions. Briefly, samples and cytokine standards were processed using the kit-specific assay procedure, and absorbance was measured at 450 nm using a microplate reader. Cytokine concentrations were determined from the corresponding standard curves and expressed as pg/mL. Measurements were obtained from three independent biological experiments (*n* = 3). The measured concentrations were not normalised to viable cell number or total cellular protein and therefore represent unadjusted cytokine concentrations in the collected culture supernatants.

### 2.8. Flow Cytometric Assessment of Mitochondrial Membrane Potential

Changes in mitochondrial membrane potential (ΔΨm) were assessed using the MitoProbe JC-1 Assay Kit (Catalogue No. M34152; Thermo Fisher Scientific, Waltham, MA, USA) and flow cytometry. 4T1 cells were exposed for 48 h to vehicle control, BA (30 µM), DOX (1.5 µM), or BA + DOX (30 + 1.5 µM), using the mechanistic treatment conditions defined in Section 2.3. Following treatment, cells were incubated with JC-1 dye (2 µg/mL) at 37 °C for 20 min in the dark, washed twice with PBS and immediately subjected to flow cytometric analysis using a FACSCalibur flow cytometer (BD Biosciences, San Jose, CA, USA).

Green fluorescence corresponding to JC-1 monomers was detected in the FL1 channel (530/30 nm), while red fluorescence corresponding to JC-1 aggregates was detected in the FL2 channel (585/42 nm). A total of 10,000 events were acquired per sample and data was analysed using FlowJo software (BD Life Sciences, Ashland, OR, USA). Mitochondrial membrane potential was quantified as the ratio of red-to-green fluorescence intensity (FL2/FL1), with a decrease in this ratio reflecting mitochondrial membrane depolarisation.

### 2.9. Quantification of Caspase-3/7 Activity

Caspase-3/7 activity was quantified using the Caspase-Glo^®^ 3/7 Assay (Promega Corporation, Madison, WI, USA) according to the manufacturer’s instructions. 4T1 cells were seeded in opaque white 96-well plates at a density of 1 × 10^4^ cells/well and allowed to attach overnight. Cells were then exposed for 48 h to vehicle control, BA (30 µM), DOX (1.5 µM), or BA + DOX (30 + 1.5 µM). Following treatment, an equal volume of Caspase-Glo^®^ 3/7 reagent was added directly to each well without removing the culture medium. The plates were gently mixed and incubated for 60 min at room temperature in the dark.

Luminescence was measured using a Synergy HTX multimode microplate reader (BioTek Instruments, Winooski, VT, USA). To account for treatment-related differences in viable cell abundance, Caspase-3/7 luminescence was normalised to the corresponding viable cell measurements obtained from parallel MTT assays performed under the same treatment conditions Normalised Caspase-3/7 activity was expressed as a fold change relative to the vehicle-treated control group, which was assigned a value of 1.0.

### 2.10. RT-qPCR Profiling of Apoptosis-Related Genes

Total RNA was isolated from 4T1 cells after 48 h of exposure to vehicle control, BA (30 µM), DOX (1.5 µM), or BA + DOX (30 + 1.5 µM) using TRIzolTM Reagent (InvitrogenTM, Thermo Fisher Scientific, Waltham, MA, USA) according to the manufacturer’s instructions. RNA concentration and purity were assessed using a NanoDropTM 2000 spectrophotometer (Thermo Fisher Scientific, Waltham, MA, USA) based on the absorbance ratio A260/A280. Only RNA samples with A260/A280 ratios between 1.8 and 2.0 were used for subsequent analyses.

First-strand cDNA was synthesised from 1 µg of total RNA using the High-Capacity cDNA Reverse Transcription Kit (Applied Biosystems, Foster City, CA, USA) according to the manufacturer’s instructions. Quantitative real-time PCR was performed using PowerUpTM SYBRTM Green Master Mix (Applied Biosystems, Foster City, CA, USA) on a QuantStudioTM 5 Real-Time PCR System (Applied Biosystems, Foster City, CA, USA). Each 20 µL reaction contained 10 µL of SYBR Green Master Mix, primers at a final concentration of 0.4 µM each, 2 µL of cDNA template and nuclease-free water. The amplification protocol consisted of an initial denaturation at 95 °C for 2 min, followed by 40 cycles of denaturation at 95 °C for 15 s and annealing/extension at 60 °C for 30 s. Melt-curve analysis was performed after amplification, and only reactions showing a single predominant melting peak without additional amplification peaks were included in the expression analysis.

Relative mRNA expression levels of the murine apoptosis-related genes *Bax*, *Bcl2*, *Casp3*, and *Casp9* were normalised to Gapdh as the endogenous reference gene and calculated using the 2^−ΔΔCt^ method, with the vehicle-treated control group used as calibrator. Three independent biological experiments were performed, and each biological sample was analysed in three technical qPCR replicates. Technical-replicate Ct values were inspected for consistency and averaged to obtain a single Ct value for each biological replicate. Relative-expression values were subsequently calculated separately for each biological replicate, and statistical analyses were performed using these biological replicate-level values rather than the individual technical replicates. The primer sequences are provided in Table 1.

### 2.11. Fluorescence-Based Live/Dead Cell Assessment

After 48 h of exposure to vehicle control, BA (30 µM), DOX (1.5 µM), or BA + DOX (30 + 1.5 µM), the status of live/dead cells was assessed by dual-fluorescence staining of Calcein-AM/PI. 4T1 cells were seeded in 24-well plates, allowed to attach overnight and subsequently exposed to the indicated treatments. After treatment, cells were washed twice with PBS and incubated with Calcein-AM and PI staining solution at 37 °C in the dark according to the manufacturer’s instructions. Calcein-AM-positive cells exhibiting green fluorescence were classified as viable, whereas PI-positive cells exhibiting red fluorescence were classified as cells with compromised plasma membrane integrity.

Three independent biological experiments (*n* = 3) were performed, with three replicate wells analysed per treatment condition in each experiment. Five randomly selected, non-overlapping microscopic fields were acquired from each well using identical imaging settings across all experimental groups. Live cells and PI-positive cells were quantified using ImageJ software (version 1.54; National Institutes of Health, Bethesda, MD, USA), and each population was expressed as a percentage of the total number of cells counted.

### 2.12. DAPI-Based Assessment of Nuclear Morphology

After 48 h of exposure to vehicle control, BA (30 µM), DOX (1.5 µM), or BA + DOX (30 + 1.5 µM), 4T1 cells were washed with PBS and fixed with 4% paraformaldehyde for 15 min at room temperature. After fixation, cells were washed with PBS and incubated with DAPI staining solution for 15 min at room temperature in the dark. Stained nuclei were examined using a fluorescence microscope equipped with an appropriate DAPI filter set (approximately 358/461 nm excitation/emission).

Nuclear morphological alterations associated with apoptosis were evaluated on the basis of chromatin condensation, nuclear shrinkage, nuclear fragmentation, and apoptotic body formation. Images were analysed using ImageJ software (version 1.54; National Institutes of Health, Bethesda, MD, USA). Nuclei displaying these apoptosis-associated morphological features were counted together with the total number of nuclei in each field. The percentage of nuclei with apoptosis-associated morphology was calculated as follows: (number of nuclei with apoptosis-associated morphology/total number of nuclei counted) × 100. Three independent biological experiments (*n* = 3) were performed, with three replicate wells analysed per treatment condition in each experiment. Five randomly selected, non-overlapping microscopic fields were examined per well using identical imaging settings across all experimental groups. Field- and well-level measurements were averaged within each biological experiment to obtain one biological replicate-level value for statistical analysis.

### 2.13. Target Prediction, Functional Enrichment, and PPI Network Analysis

A database-based bioinformatics workflow was conducted to identify predicted molecular targets shared among genes associated with BA, DOX, and breast cancer and to explore their potential functional associations with apoptosis-related signalling. The hypothetical targets for BA and DOX were retrieved from SwissTargetPrediction, SuperPred, and PharmMapper, producing 387 BA-associated and 452 DOX-associated targets, respectively. Breast cancer-associated genes (*n* = 1284) were obtained from GeneCards, OMIM, and DisGeNET. Following removal of duplicate entries and intersection analysis, 94 predicted common targets were identified and subjected to enrichment analyses of the GO and KEGG pathway. Because no transcriptome-wide differential expression analysis was performed, these targets were considered predicted common targets rather than differentially expressed genes.

Because the experimental studies were performed with murine 4T1 cells, mouse gene identifiers were assigned to their corresponding human orthologs using the NCBI HomoloGene database before network analysis to ensure compatibility with the Homo sapiens reference data set used in STRING. PPI analysis was performed using STRING (version 12.0; Homo sapiens) with a minimum interaction confidence score of 0.70. Of the 94 predicted common targets, interconnected proteins associated with apoptosis that met the predefined STRING confidence threshold were retained for focused PPI analysis. The resulting network comprised 11 interconnected proteins and was imported into Cytoscape (version 3.10.2) for visualisation and topological analysis. The hub proteins were classified according to network connectivity (degree) and betweenness centrality (BC).

GO enrichment analyses encompassing Biological Process (BP), Cellular Component (CC), and Molecular Function (MF), together with KEGG pathway enrichment analysis, were performed using the STRING enrichment module. Enrichment terms with a Benjamini–Hochberg-adjusted false discovery rate (FDR) < 0.05 were considered statistically significant. These analyses were considered exploratory and were used to identify putative functional associations rather than experimentally validated molecular mechanisms.

### 2.14. Statistical Analysis and Evaluation of Drug Interactions

All quantitative data were obtained from three independent biological experiments (*n* = 3) and are presented as mean ± standard deviation (SD). The technical replicates were averaged within each biological experiment and were not treated as independent observations. Statistical analyses were performed with GraphPad Prism version 9.0 (GraphPad Software, San Diego, CA, USA). Comparisons among multiple treatment groups were made using one-way analysis of variance (ANOVA) followed by Tukey’s multiple comparison test, while comparisons between two independent groups were made using an unpaired two-tailed Student’s *t*-test. A two-sided *p* < 0.05 was considered statistically significant.

The MTT data were normalised to the vehicle-treated control and expressed as percentage cell viability, and the resulting dose–response data were used to determine the IC50 values. The selectivity indices (SI) were calculated as IC_50_ (HaCaT)/IC_50_ (4T1). Drug interactions were independently evaluated using the Chou–Talalay CI, HSA, and Bliss independence models. For categorical interpretation, CI < 0.8 was classified as synergistic, 0.8 ≤ CI ≤ 1.2 as near-additive, and CI > 1.2 as antagonistic; CI = 1 represented theoretical additivity. Differences among the three interaction models were interpreted as model-dependent effects.

For flow-cytometric, cytokine, Caspase-3/7, RT-qPCR, and fluorescence-imaging analyses, statistical comparisons were based on biological replicate-level values. Microscopic fields and replicate wells were treated as subsamples rather than independent observations. Total apoptosis was calculated as the sum of the early and late-apoptotic populations, and relative gene expression was determined using the 2^−ΔΔCt^ method. For bioinformatic enrichment analyses, Benjamini–Hochberg-adjusted FDR < 0.05 was considered significant, while the topology of the PPI network was descriptively evaluated using degree and BC.

## 3. Results

### 3.1. BA and DOX Reduce Cell Viability in a Concentration- and Time-Dependent Manner

The MTT analysis demonstrated concentration- and time-dependent reductions in cell viability after BA and DOX treatment in 4T1 and HaCaT cells (Figure 1). In 4T1 cells, increasing BA concentrations progressively reduced cell viability, with a more pronounced reduction after 48 h than after 24 h (Figure 1A). The 48 h IC_50_ value for BA was 30.2 µM (95% CI: 28.3–32.1 µM). In HaCaT cells, BA produced a comparatively smaller reduction in viability, particularly after 24 h, when cell viability remained above 50% throughout the tested concentration range (5–50 µM). After 48 h, the IC_50_ value for BA in HaCaT cells was 38.6 µM (95% CI: 36.4–40.8 µM) (Figure 1B).

DOX produced a marked concentration- and time-dependent reduction in cell viability in both cell lines (Figure 1C,D). After 48 h, the IC_50_ values for DOX were 1.8 µM (95% CI: 0.6–3.1 µM) in 4T1 cells and 2.8 µM (95% CI: 1.2–4.4 µM) in HaCaT cells. Consistent with the concentration–response profiles, the IC_50_ values at 48 h of BA and DOX were higher in HaCaT cells than in 4T1 cells, indicating comparatively greater cytotoxicity towards 4T1 cells under experimental conditions (Figure 1E).

Based on the 48 h IC_50_ values, the selectivity index (SI = IC_50_ HaCaT/IC_50_ 4T1) was 1.28 for BA and 1.56 for DOX (Figure 1F). These values indicate modest differential cytotoxicity toward 4T1 cells rather than strong cancer-cell selectivity.

### 3.2. BA–DOX Interactions Show Concentration- and Model-Dependent Effects

**Figure 2:**
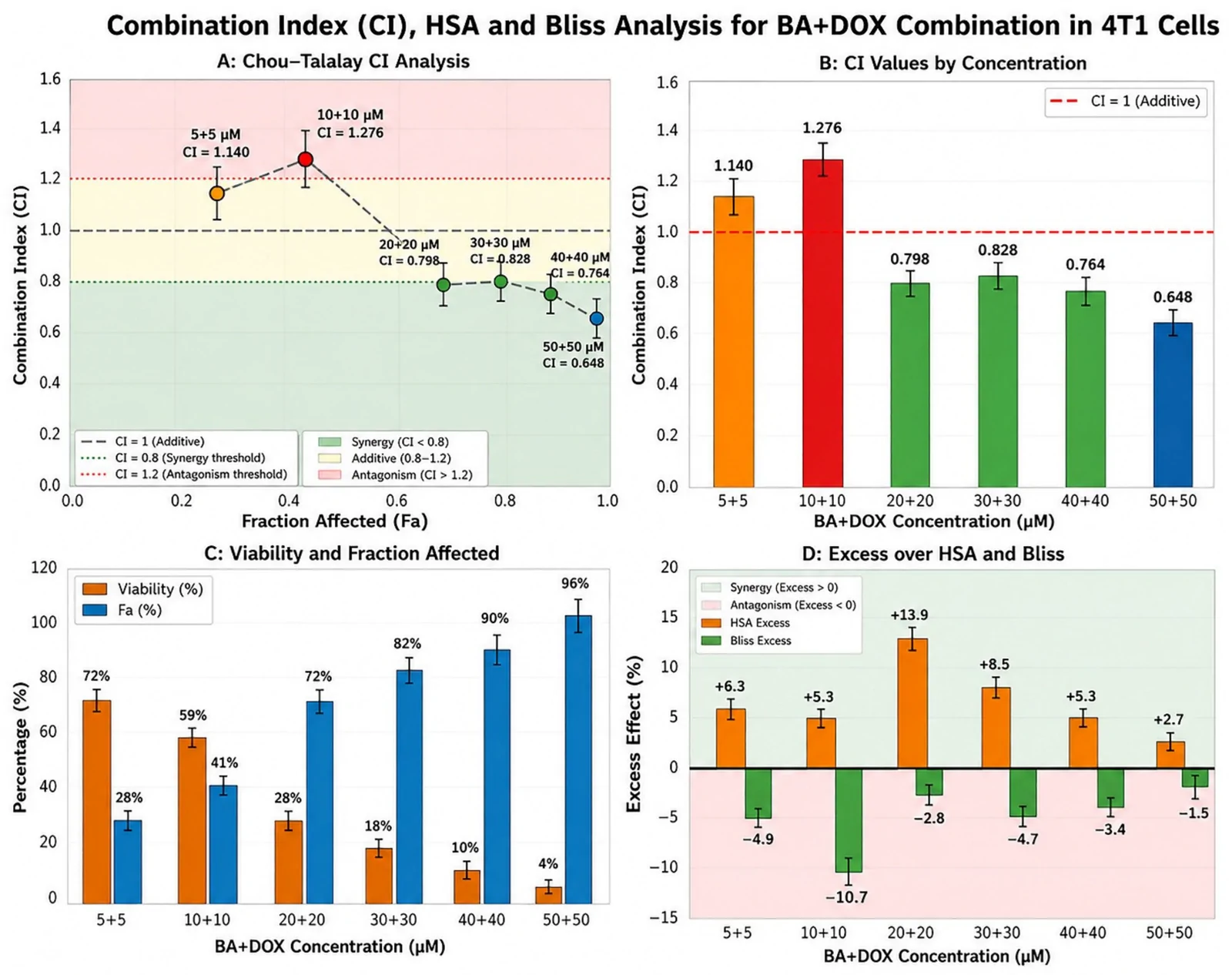
Drug-interaction analysis of BA and DOX in 4T1 cells. (**A**) Chou–Talalay CI analysis, with CI < 0.8 classified as synergistic, 0.8 ≤ CI ≤ 1.2 as near-additive, and CI > 1.2 as antagonistic. The 5 + 5 μM and 30 + 30 μM combinations were near-additive, the 10 + 10 μM combination was antagonistic, and the 20 + 20, 40 + 40, and 50 + 50 μM combinations were classified as synergistic, with the lowest CI observed at 50 + 50 μM (CI = 0.648). (**B**) CI values for the tested BA + DOX concentration pairs; the dashed line indicates the theoretical additivity reference at CI = 1. (**C**) Cell viability and affected fraction (Fa) across combination concentrations. (**D**) Excess-over-HSA and Bliss analyses. HSA excess values were positive, whereas Bliss excess values were negative, indicating model-dependent differences in drug-interaction assessment. Overall, BA–DOX interactions were concentration- and model-dependent rather than uniformly synergistic.

The interaction between BA and DOX was evaluated after 48 h of exposure using fixed 1:1 concentration pairs and the Chou–Talalay, HSA, and Bliss independence models (Figure 2). According to the operational CI classification criteria described in the Methods, the 5 + 5 μM combination showed a near-additive interaction (CI = 1.140), while 10 + 10 μM showed antagonism (CI = 1.276). At 20 + 20 μM, the CI decreased to 0.798, which was slightly below the predefined threshold of 0.8 and was therefore classified as synergistic, with cell viability of 28% and an Fa of 0.72. The 30 + 30 μM combination was classified as near-additive (CI = 0.828), whereas 40 + 40 and 50 + 50 μM showed synergistic interactions, with CI values of 0.764 and 0.648, respectively. The lowest CI was observed at 50 + 50 μM, corresponding to 4% cell viability and an Fa of 0.96 (Figure 2A–C).

The HSA analysis yielded positive excess values in all combinations tested (+2.7% to +13.9%), with the highest value observed at 20 + 20 μM (+13.9%). In contrast, Bliss analysis produced negative excess values across all concentration pairs (−1.5% to −10.7%), indicating that the observed combined effects did not exceed Bliss-predicted responses (Figure 2D). Thus, the interaction profile was model-dependent: HSA yielded positive excess effects relative to the highest single-agent reference, Bliss yielded negative excess values, and the Chou–Talalay analysis classified only selected concentration pairs as synergistic. These findings therefore did not indicate uniform BA–DOX synergy across the tested concentration range or analytical models.

The 30 μM BA + 1.5 μM DOX condition used for subsequent phenotypic-characterisation experiments was not included in this fixed-ratio drug-interaction series. Therefore, no CI-, HSA-, or Bliss-based synergy classification was assigned to this treatment condition.

### 3.3. BA and DOX Increase Apoptosis Cell Death in 4T1 Cells

**Figure 3:**
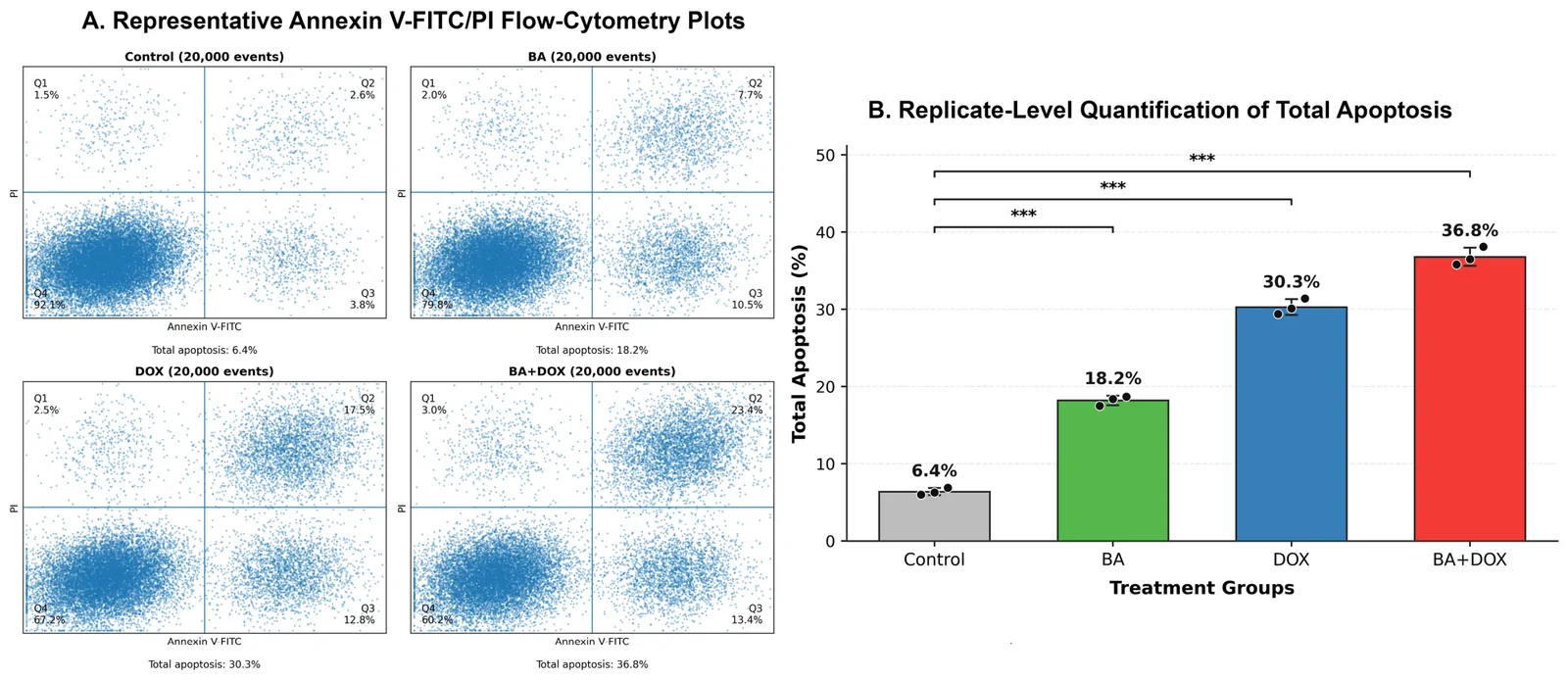
Effect of BA and DOX on apoptosis in 4T1 breast cancer cells determined by Annexin V-FITC/PI flow cytometry. (**A**) Representative flow-cytometry dot plots showing the distribution of viable and apoptotic cell populations after 48 h of treatment with BA (30 μM), DOX (1.5 μM), or their combination (BA + DOX; 30 + 1.5 μM). A total of 20,000 events were acquired for each sample. Cell populations were classified into four quadrants: Q4 (Annexin V^−^/PI^−^), viable cells; Q3 (Annexin V^+^/PI^−^), early apoptotic cells; Q2 (Annexin V^+^/PI^+^), late apoptotic/secondary necrotic cells; and Q1 (Annexin V^−^/PI^+^), necrotic cells. Total apoptosis was calculated as the sum of the early- and late-apoptotic populations (Q3 + Q2). The total apoptotic fractions shown in the representative plots were 6.4% in the vehicle-treated control group, 18.2% in the BA group, 30.3% in the DOX group, and 36.8% in the BA + DOX group. (**B**) Replicate-level quantification of total apoptosis. Bars represent the mean ± SD of three independent biological experiments (*n* = 3), and individual dots represent the values obtained from each biological replicate. Statistical comparisons were performed using one-way ANOVA followed by Tukey’s multiple-comparison test. *** *p* < 0.001 versus the vehicle-treated control group.

Annexin V-FITC/PI flow-cytometric analysis demonstrated treatment-associated increases in apoptotic cell populations (Figure 3A). Replicate-level quantification showed that mean total apoptosis increased from 6.4% in the vehicle-treated control group to 18.2% with BA, 30.3% with DOX, and 36.8% with BA + DOX (Figure 3B). All treatment groups showed significantly higher total apoptosis than the vehicle-treated control group (*** *p* < 0.001). In the representative plots, early apoptotic cells increased from 3.8% in the vehicle-treated control group to 10.5%, 12.8%, and 13.4% in the BA, DOX, and BA + DOX groups, respectively. Late apoptotic cells increased from 2.6% to 7.7%, 17.5%, and 23.4%, respectively. The BA + DOX group exhibited the highest mean total apoptotic fraction among the examined groups, while its representative plot also showed the highest late-apoptotic fraction.

### 3.4. Cell Cycle Redistribution After BA and DOX Treatment

**Figure 4:**
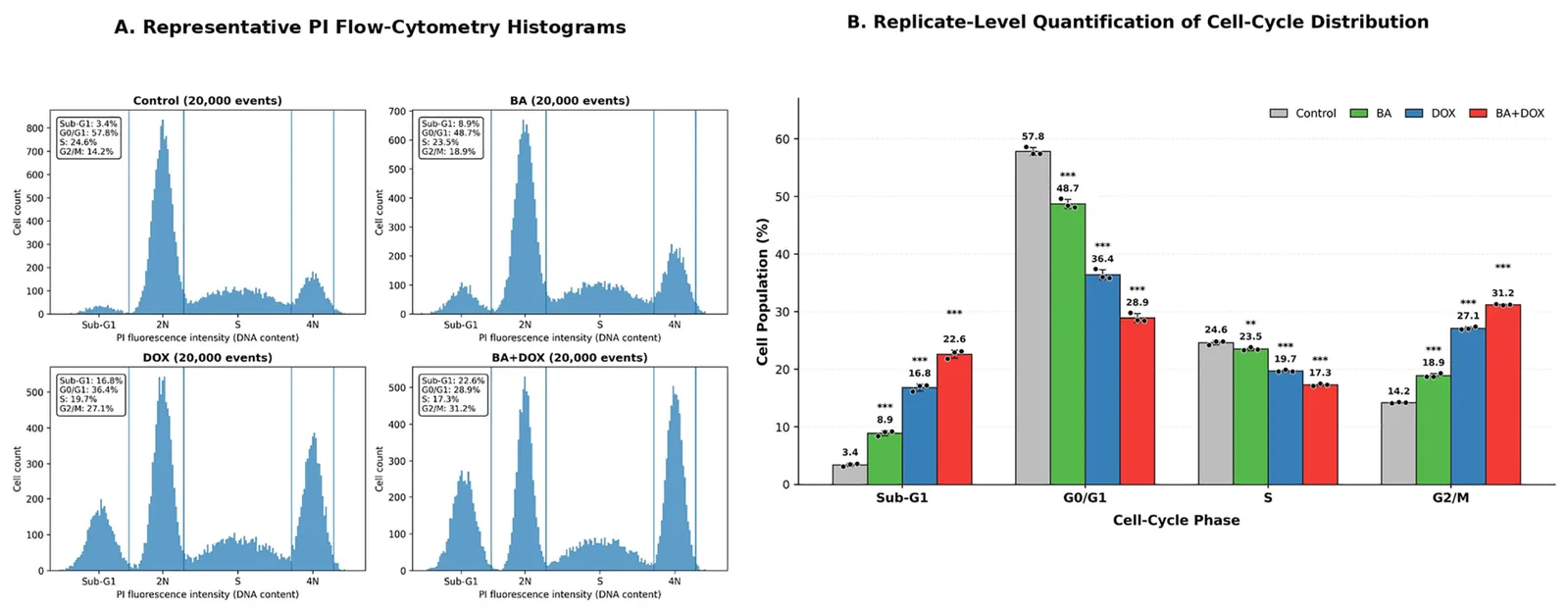
Effects of BA and DOX on cell-cycle distribution in 4T1 breast cancer cells. (**A**) Representative DNA-content histograms obtained by PI staining and flow cytometry after 48 h of treatment with BA (30 μM), DOX (1.5 μM), or their combination (BA + DOX; 30 + 1.5 μM). A total of 20,000 events were acquired per sample. Cell populations were classified as Sub-G1 (hypodiploid DNA population associated with DNA fragmentation), G0/G1 (2N DNA content), S phase (DNA synthesis), and G2/M (4N DNA content). (**B**) Quantification of the percentages of cells within each cell-cycle phase. Data are presented as the mean ± SD of three independent experiments, with individual data points representing separate experiments. Statistical comparisons were performed separately for each phase using one-way ANOVA followed by Tukey’s multiple-comparisons test. ** *p* < 0.01, and *** *p* < 0.001 versus the corresponding control group.

PI-based cell-cycle analysis demonstrated treatment-associated alterations in cell-cycle distribution (Figure 4). Representative PI-based DNA-content histograms are presented in Figure 4A, whereas replicate-level quantification of the cell-cycle populations is shown in Figure 4B. In the control group, cells were predominantly distributed in the G0/G1 phase (57.8%), followed by the S (24.6%) and G2/M (14.2%) phases, with a Sub-G1 fraction of 3.4%. BA treatment reduced the G0/G1 population to 48.7%, accompanied by increases in the G2/M (18.9%) and Sub-G1 (8.9%) fractions and a modest reduction in the S-phase population to 23.5%. DOX treatment further decreased the G0/G1 population to 36.4%, while increasing the G2/M and Sub-G1 fractions to 27.1% and 16.8%, respectively, and reducing the S-phase fraction to 19.7%. The BA + DOX combination produced the largest numerical redistribution, with the G0/G1 and S-phase fractions decreasing to 28.9% and 17.3%, respectively, and the G2/M and Sub-G1 fractions increasing to 31.2% and 22.6%, respectively. As shown in Figure 4B, all treatment groups exhibited significant increases in the Sub-G1 and G2/M populations and significant decreases in the G0/G1 population compared with the control group (all *p* < 0.001). The S-phase population was also significantly decreased following BA (** *p* < 0.01), DOX (*** *p* < 0.001), and BA + DOX (*** *p* < 0.001) treatment. The marked increase in the Sub-G1 population shown in Figure 4 was consistent with the higher apoptotic fraction observed by Annexin V-FITC/PI analysis, although Sub-G1 accumulation alone does not provide definitive evidence of apoptosis.

### 3.5. Modulation of Cytokine Levels by BA and DOX

**Figure 5:**
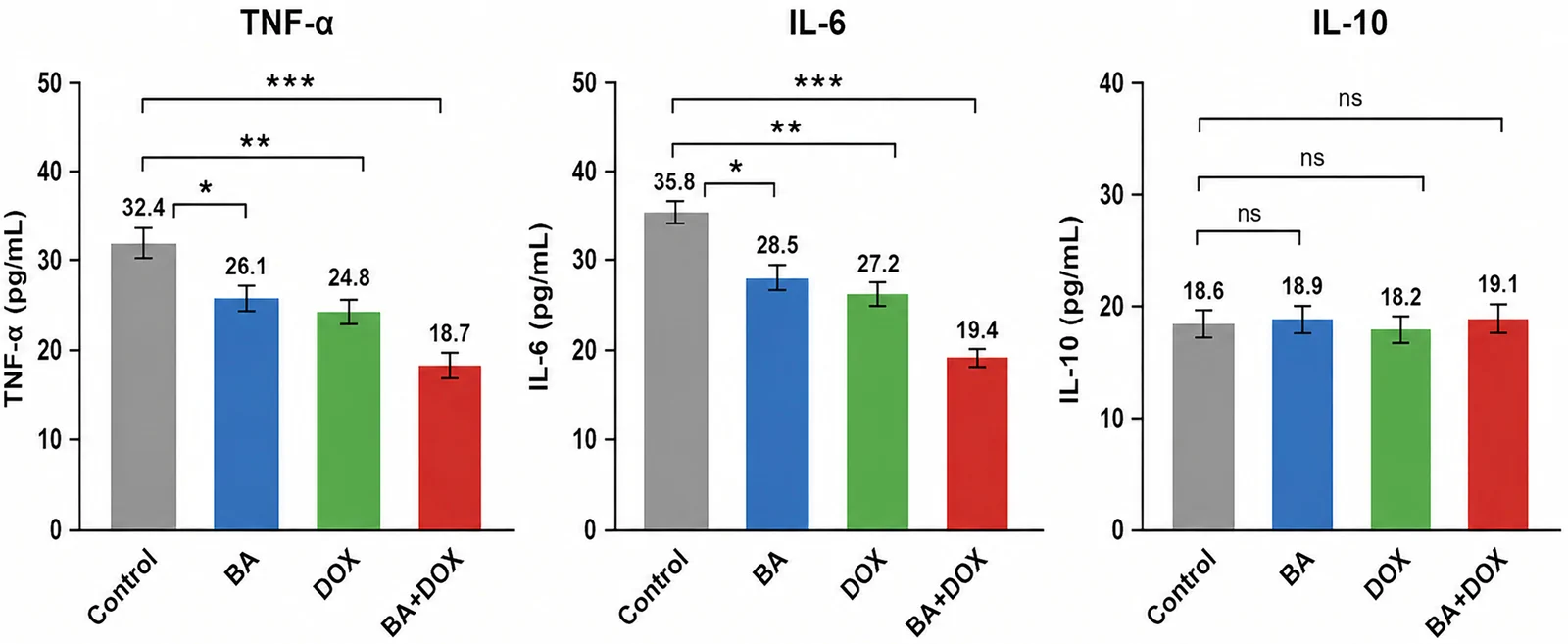
Effect of BA and DOX on inflammatory cytokine levels in 4T1 breast cancer cells. The concentrations of the pro-inflammatory cytokines TNF-α and IL-6 and the anti-inflammatory cytokine IL-10 were determined in the culture supernatants after 48 h of treatment with BA (30 μM), DOX (1.5 μM), or their combination (BA + DOX; 30 + 1.5 μM) using ELISA. Data are presented as mean ± SD of three independent experiments. The measured TNF-α and IL-6 concentrations were significantly lower than those in the vehicle-treated control, with the lowest measured concentrations observed in the BA + DOX group (*** *p* < 0.001 vs. control). These cytokine concentrations were not normalised to viable cell number or total cellular protein and should therefore be interpreted as unadjusted supernatant concentrations. In contrast, IL-10 levels did not differ significantly between the experimental groups. Statistical significance versus the control group is indicated as *p* < 0.05 (*), *p* < 0.01 (**), and *p* < 0.001 (***); ns, not significant.

Treatment with BA and DOX significantly altered the levels of the pro-inflammatory cytokines TNF-α and IL-6 in 4T1 cells (Figure 5). TNF-α levels decreased from 32.4 pg/mL in the control group to 26.1 pg/mL after BA treatment (* *p* < 0.05) and 24.8 pg/mL after DOX treatment (** *p* < 0.01). The lowest TNF-α level was observed in the BA + DOX group (18.7 pg/mL; *** *p* < 0.001 vs. control).

A similar pattern was observed for IL-6. Compared to the control level of 35.8 pg/mL, IL-6 decreased to 28.5 pg/mL after BA treatment (* *p* < 0.05), 27.2 pg/mL after DOX treatment (** *p* < 0.01) and 19.4 pg/mL after BA + DOX treatment (*** *p* < 0.001 vs. control). Thus, the combination group exhibited the lowest measured levels of both TNF-α and IL-6.

In contrast, IL-10 levels remained comparable between the control (18.6 pg/mL), BA (18.9 pg/mL), DOX (18.2 pg/mL) and BA + DOX (19.1 pg/mL) groups, with no statistically significant differences relative to the control (*p* > 0.05) (Figure 5).

### 3.6. Depolarisation of the Mitochondrial Membrane After BA and DOX Treatment

**Figure 6:**
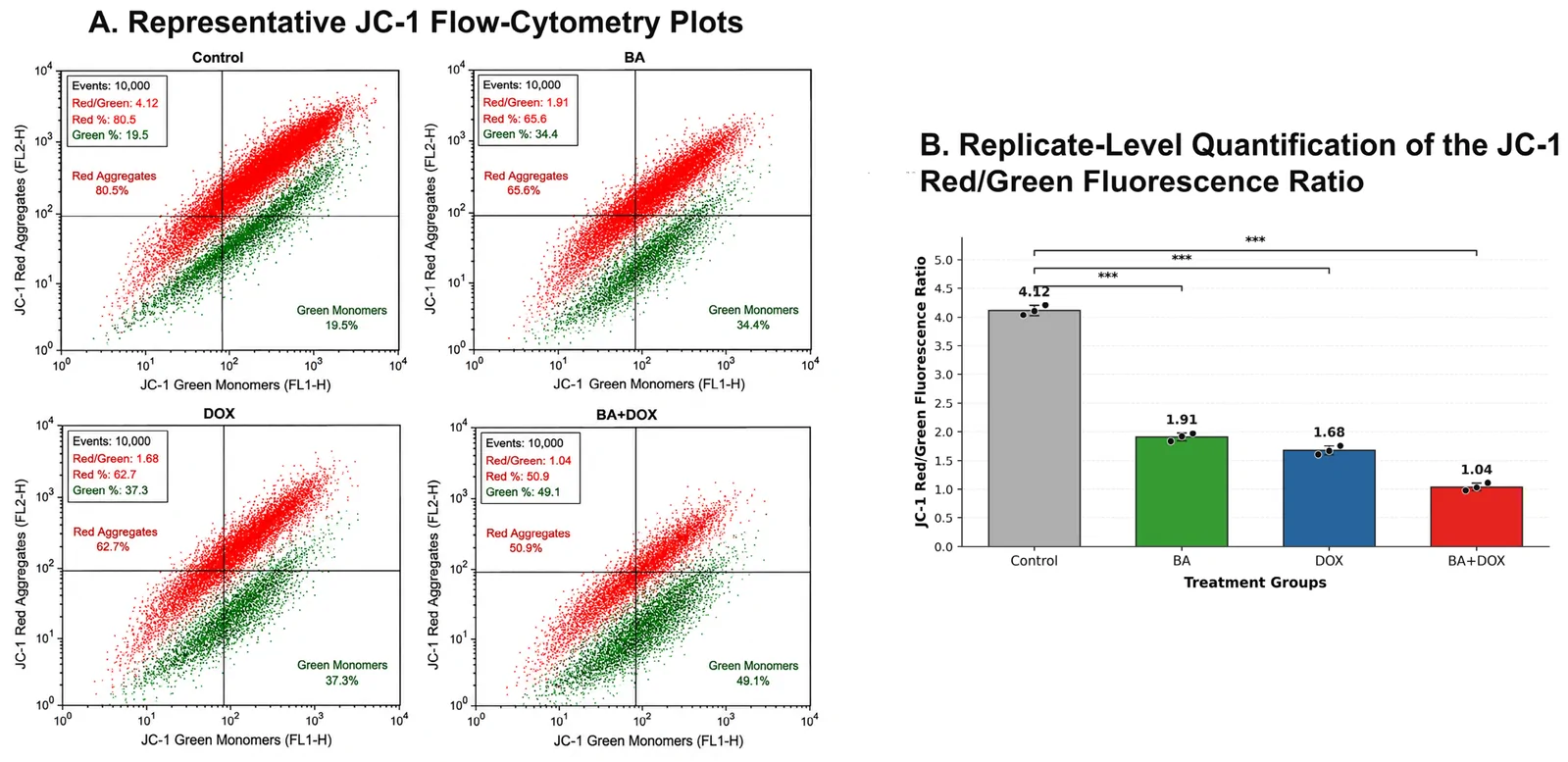
Effect of BA and DOX on mitochondrial membrane potential (ΔΨm) in 4T1 breast cancer cells determined by JC-1 staining. (**A**) Representative JC-1 flow-cytometry dot plots showing treatment-associated changes in mitochondrial membrane potential after 48 h of exposure to BA (30 μM), DOX (1.5 μM), or their combination (BA + DOX; 30 + 1.5 μM). A total of 10,000 events were acquired for each sample. Cells with preserved mitochondrial membrane potential exhibit predominant red JC-1 aggregates, whereas mitochondrial depolarisation is associated with increased green JC-1 monomers. In the representative plots, the proportion of red JC-1 aggregates decreased from 80.5% in the vehicle-treated control group to 65.6%, 62.7%, and 50.9% following BA, DOX, and BA + DOX treatment, respectively, whereas the corresponding green JC-1 monomer proportions increased from 19.5% to 34.4%, 37.3%, and 49.1%. (**B**) Replicate-level quantification of the JC-1 red/green fluorescence ratio. The mean ratio decreased from 4.12 in the vehicle-treated control group to 1.91, 1.68, and 1.04 in the BA, DOX, and BA + DOX groups, respectively. Bars represent the mean ± SD of three independent biological experiments (*n* = 3), and individual dots represent the values obtained from each biological replicate. Statistical comparisons were performed using one-way ANOVA followed by Tukey’s multiple-comparison test. *** *p* < 0.001 versus the vehicle-treated control group.

JC-1 analysis demonstrated a treatment-associated decrease in mitochondrial membrane potential (ΔΨm) in 4T1 cells (Figure 6). Replicate-level quantification showed that the mean red/green fluorescence ratio decreased from 4.12 in the vehicle-treated control group to 1.91 after BA treatment and 1.68 after DOX treatment (Figure 6B). The BA + DOX group exhibited the lowest mean red/green fluorescence ratio (1.04), corresponding to an approximately 74.8% reduction relative to the vehicle-treated control. All treatment groups showed significantly lower red/green fluorescence ratios than the vehicle-treated control group (*** *p* < 0.001). In the representative plots, the proportion of JC-1 red aggregates decreased from 80.5% in the vehicle-treated control group to 65.6%, 62.7%, and 50.9% in the BA, DOX, and BA + DOX groups, respectively, whereas the corresponding green monomer populations increased from 19.5% to 34.4%, 37.3%, and 49.1% (Figure 6A). These findings indicate treatment-associated mitochondrial membrane depolarisation, with the lowest mean red/green fluorescence ratio observed in the BA + DOX group. However, the JC-1 findings alone were not interpreted as evidence establishing activation of the intrinsic mitochondrial apoptotic pathway.

### 3.7. Enhanced Caspase-3/7 Activity Following BA and DOX Treatment

**Figure 7:**
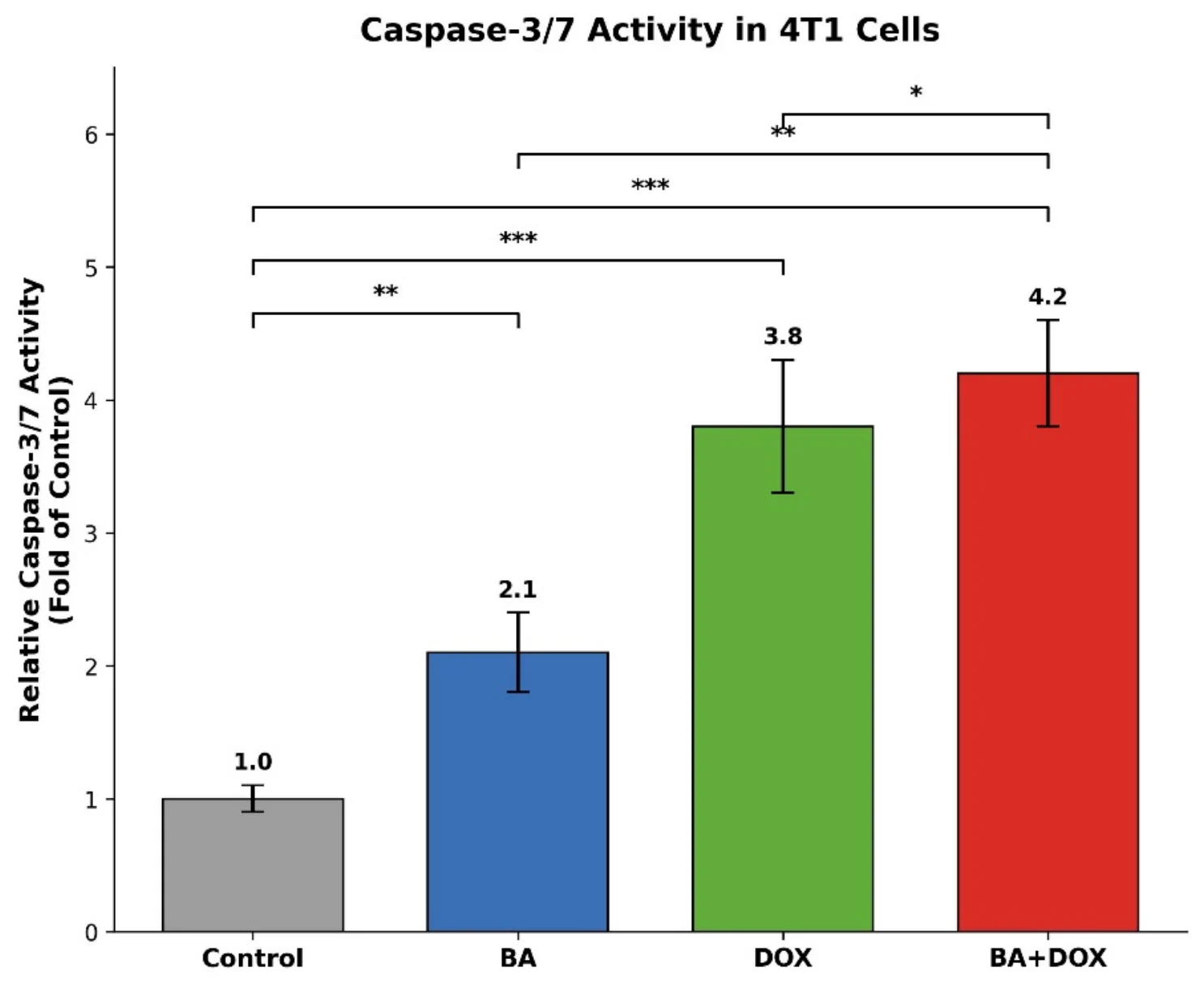
Effect of BA and DOX on caspase-3/7 activity in 4T1 breast cancer cells. Relative caspase-3/7 activity was measured using the Caspase-Glo^®^ 3/7 luminescent assay after 48 h of treatment with BA (30 μM), DOX (1.5 μM), or their combination (BA + DOX; 30 + 1.5 μM). Caspase-3/7 activity is expressed as a fold change relative to the vehicle-treated control, which was normalised to 1.0. BA treatment increased caspase-3/7 activity 2.1 times (** *p* < 0.01 vs. control), while DOX increased activity 3.8 times (*** *p* < 0.001 vs. control). The combination of BA + DOX produced the highest caspase-3/7 activity (4.2 times; *** *p* < 0.001 vs. control), which was significantly higher than that observed with BA alone (*** *p* < 0.001) and DOX alone (* *p* < 0.05). Data are presented as mean ± SD from three independent experiments. Statistical significance is indicated as *p* < 0.05 (*), *p* < 0.01 (**), and *p* < 0.001 (***).

MTT-normalised caspase-3/7 activity increased after BA and DOX treatment, with the highest normalised activity observed in the combination group (Figure 7). The Caspase-Glo^®^ 3/7 luminescence signal was normalised to the corresponding viable-cell measurement obtained from parallel MTT assays performed under the same treatment conditions. Compared to the vehicle-treated control (1.0 ± 0.1-fold), BA treatment increased normalised caspase-3/7 activity to 2.1 ± 0.3-fold (*p* < 0.01), while DOX increased activity to 3.8 ± 0.5-fold (*p* < 0.001). The combination of BA + DOX resulted in the highest normalised caspase-3/7 activity (4.2 ± 0.4-fold; *p* < 0.001 vs. control), which was significantly higher than that observed with BA alone (*p* < 0.001) and DOX alone (*p* < 0.05). These results demonstrate increased MTT-normalised caspase-3/7 activity after combined BA and DOX treatment.

### 3.8. RT-qPCR Analysis of Apoptosis-Related Gene Expression

All RNA samples included in the analysis met the predefined A260/A280 acceptance range of 1.8–2.0, and the included amplification reactions showed a single predominant melt-curve peak. Technical-replicate Ct values were averaged within each biological replicate before relative-expression calculations.

**Figure 8:**
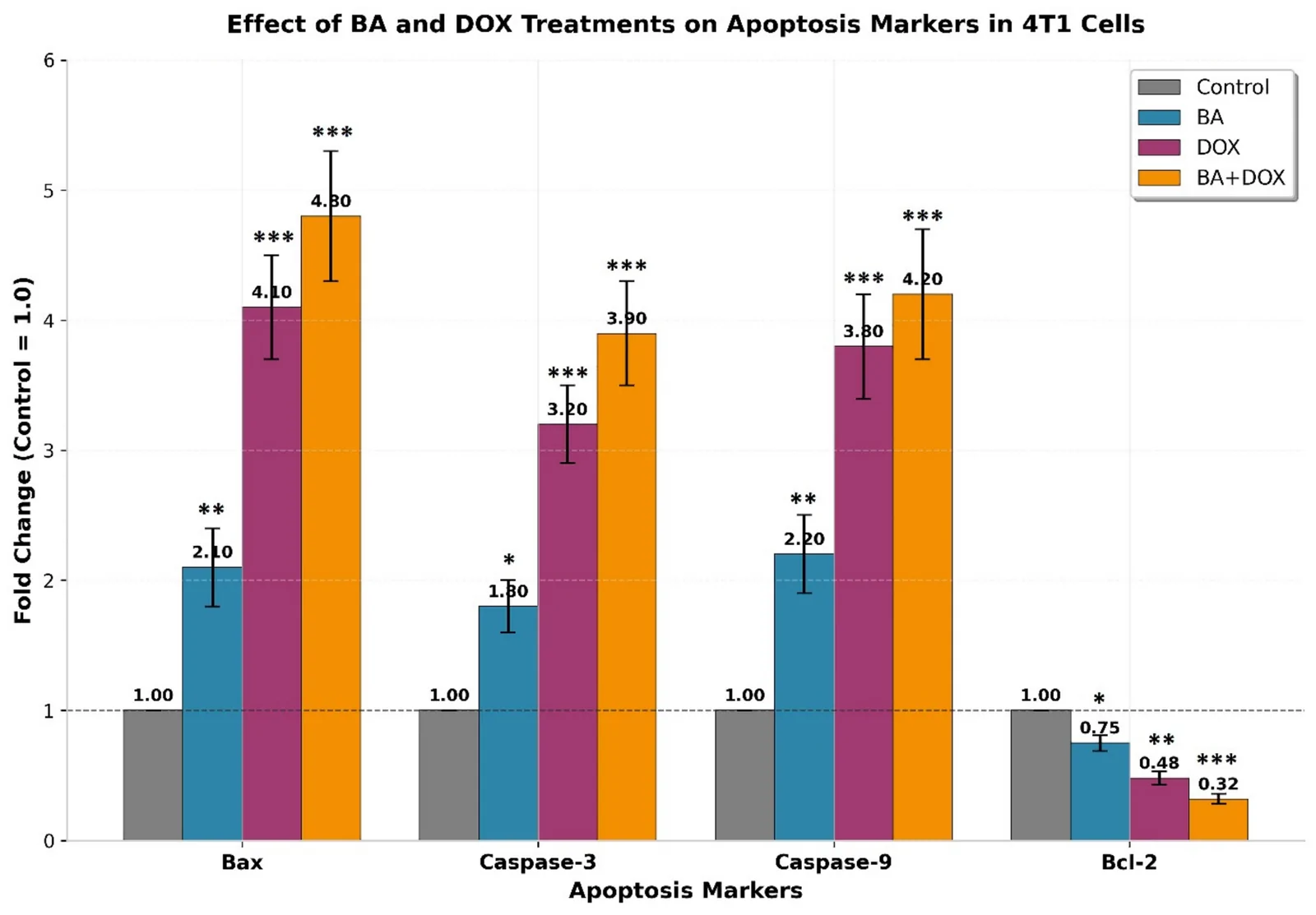
Effect of BA and DOX on the expression of genes related to apoptosis in 4T1 breast cancer cells. Relative mRNA expression levels of *Bax*, *Casp3*, *Casp9*, and *Bcl2* were determined by quantitative real-time PCR (RT-qPCR) after 48 h of treatment with BA (30 μM), DOX (1.5 μM), or their combination (BA + DOX; 30 + 1.5 μM). Gene expression levels were normalised to the reference gene Gapdh and calculated using the 2^−ΔΔCt^ method, with the vehicle-treated control group set to 1.0. Data are presented as mean ± SD of three independent experiments performed in triplicate. Statistical comparisons were performed using one-way analysis of variance (ANOVA) followed by Tukey’s multiple-comparison test. *p* < 0.05 (*), *p* < 0.01 (**) and *p* < 0.001 (***) versus the vehicle-treated control. The dashed horizontal line indicates the control reference level (fold change = 1.0).

Relative mRNA expression analysis demonstrated treatment-dependent modulation of apoptosis-related genes in 4T1 cells (Figure 8). BA treatment increased the expression of the pro-apoptotic genes *Bax* (2.1 ± 0.3-fold), *Casp3* (1.8 ± 0.2-fold), and *Casp9* (2.2 ± 0.3-fold), while reducing *Bcl2* expression to 0.75 ± 0.06-fold relative to the vehicle-treated control. DOX treatment produced greater changes, increasing the expression of *Bax* (4.1 ± 0.4-fold), *Casp3* (3.2 ± 0.3-fold), and *Casp9* (3.8 ± 0.4-fold), while decreasing the expression of *Bcl2* to 0.48 ± 0.05-fold.

The combination of BA + DOX produced the most pronounced changes, increasing *Bax*, *Casp3*, and *Casp9* expression to 4.8 ± 0.5-, 3.9 ± 0.4-, and 4.2 ± 0.5-fold, respectively, while decreasing *Bcl2* expression to 0.32 ± 0.04-fold. In general, these transcriptional changes indicate that the combination treatment was associated with the strongest shift towards a pro-apoptotic gene expression profile (Figure 8). However, these mRNA-level findings were not interpreted as direct evidence of corresponding protein-level changes or activation of the mitochondrial apoptotic pathway.

### 3.9. Bax/Bcl2 Ratio

**Figure 9:**
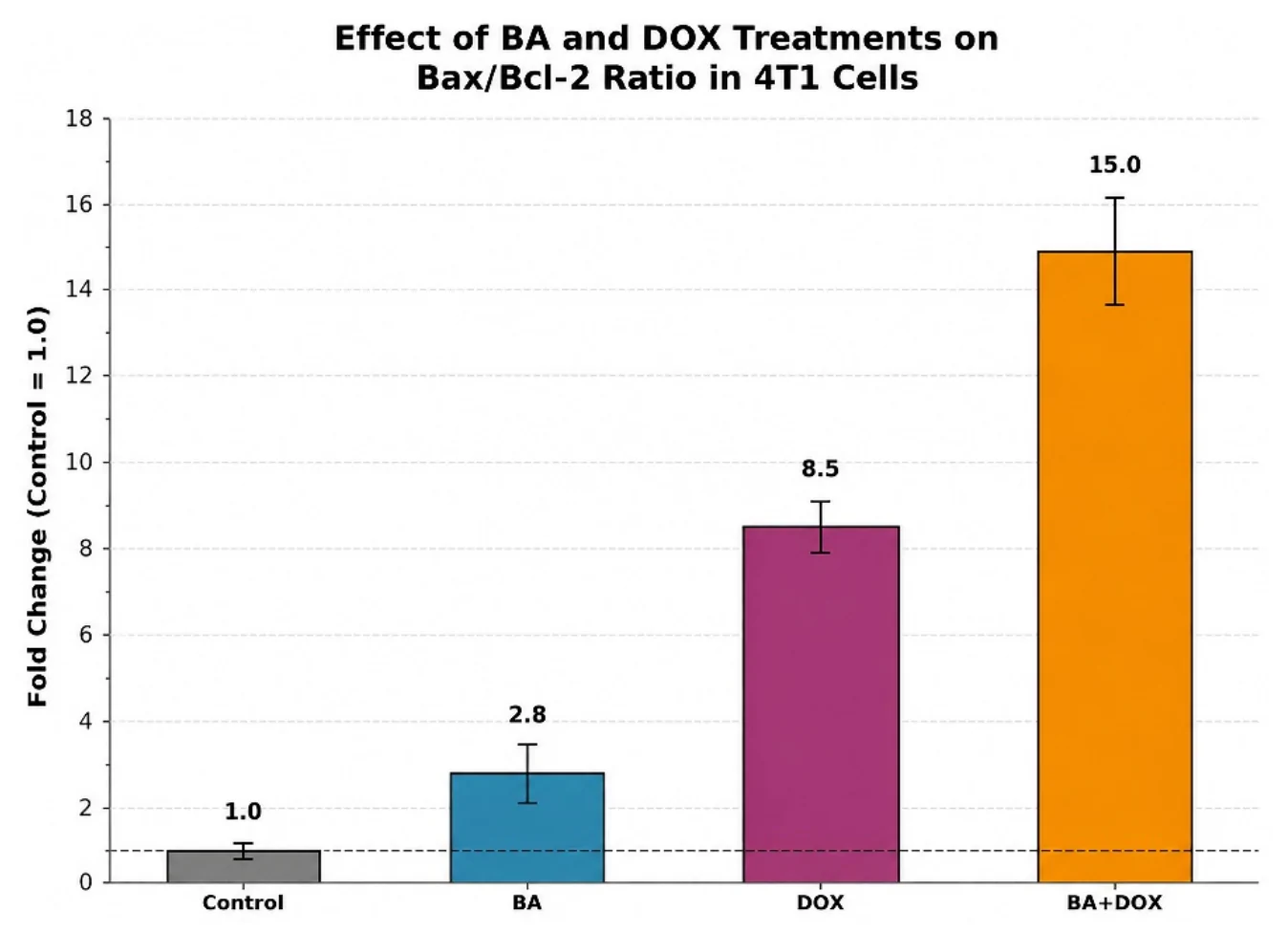
Effect of BA and DOX on the expression ratio of *Bax*/*Bcl2* mRNA in 4T1 breast cancer cells. The *Bax*/*Bcl2* ratio was calculated from the relative expression levels of mRNA determined by RT-qPCR after 48 h of treatment with BA (30 μM), DOX (1.5 μM), or their combination (BA + DOX; 30 + 1.5 μM). The ratio was calculated individually for each biological replicate prior to statistical analysis and normalised relative to the vehicle-treated control group (control = 1.0). Data are presented as mean ± SD from three independent experiments. The dashed horizontal line indicates the control reference level (fold change = 1.0).

Consistent with the individual gene expression results, the expression ratio of the *Bax*/*Bcl2* mRNA progressively increased after treatment (Figure 9). The ratio increased from 1.0 in the control group to 2.8 after BA treatment, 8.5 after DOX treatment, and 15.0 after BA + DOX treatment. The combination group therefore exhibited the highest Bax/Bcl2 ratio, consistent with a pronounced shift toward a pro-apoptotic transcriptional profile. The *Bax*/*Bcl2* ratio was calculated individually for each biological replicate before statistical analysis; therefore, the mean ± SD values represent the average of the replication-level ratios rather than the ratios calculated directly from the mean expression values of the group (Figure 9).

### 3.10. Calcein-AM/PI Live–Dead Staining Findings

**Figure 10:**
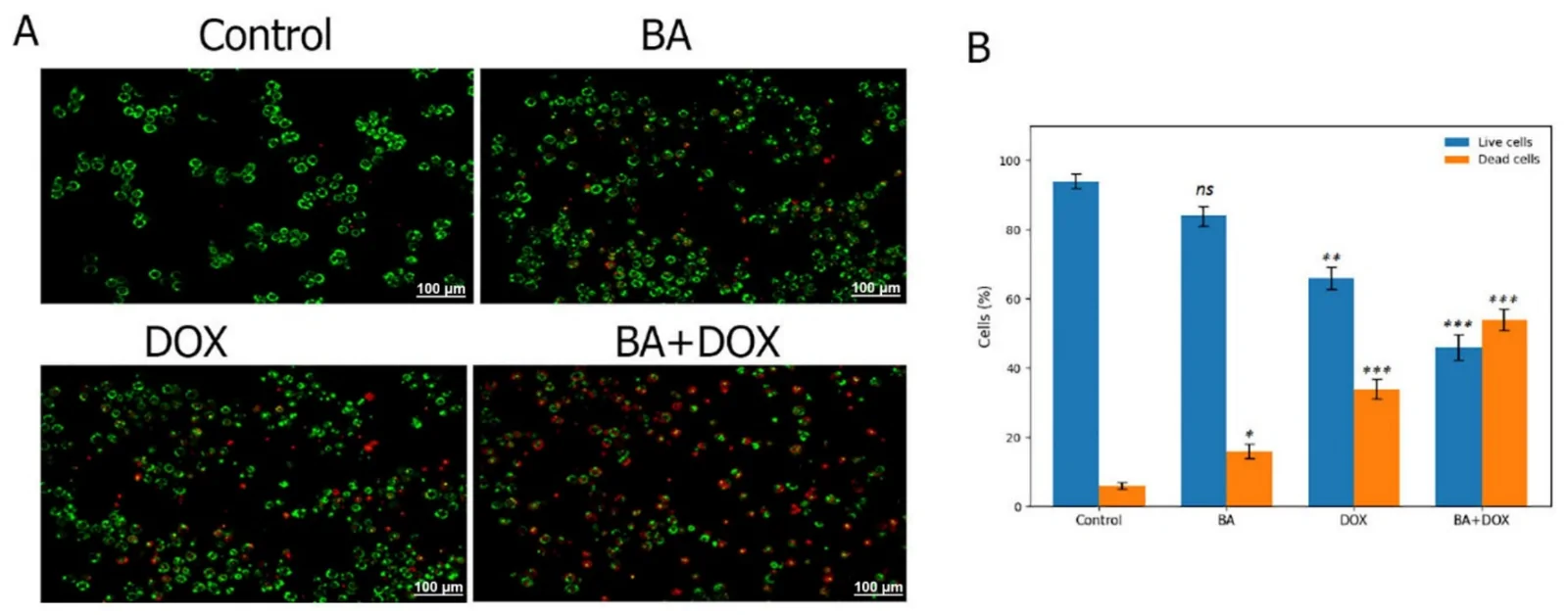
Effect of BA and DOX on cell viability in 4T1 breast cancer cells determined by live/dead Calcein-AM/PI staining. (**A**) Representative fluorescence images obtained after 48 h of treatment with BA (30 μM), DOX (1.5 μM), or their combination (BA + DOX; 30 + 1.5 μM). The control group exhibited predominantly Calcein-AM positive green fluorescence with minimal PI-positive red fluorescence. BA treatment moderately increased PI-positive cells, whereas DOX treatment reduced the proportion of Calcein-AM-positive cells and increased PI-positive cells. The BA + DOX combination produced the greatest reduction in viable cells and the highest proportion of PI-positive cells. Representative images are shown from three independent experiments performed under identical acquisition settings. Scale bar = 100 μm. (**B**) Quantitative analysis of viable cells positive for Calcein-AM and dead cells positive for PI. Cell populations were quantified from five randomly selected, non-overlapping microscopic fields per well using ImageJ software. Data are presented as mean ± SD from three independent experiments. Statistical comparisons were performed using one-way ANOVA followed by Tukey’s multiple-comparison test. *p* < 0.05 (*), *p* < 0.01 (**), and *p* < 0.001 (***) versus the vehicle-treated control. ns, not significant.

Calcein-AM/PI staining demonstrated treatment-dependent changes in 4T1 cell viability after exposure to BA and DOX (Figure 10). The control group exhibited predominantly Calcein-AM-positive green fluorescence with minimal PI-positive red fluorescence, consistent with a high proportion of viable cells. BA treatment moderately increased PI-positive cells, while Calcein-AM-positive cells remained predominant.

Quantitative analysis showed approximately 94% viable and 6% dead cells in the control group. Following BA treatment, the proportions were approximately 84% viable and 16% dead cells. DOX treatment further reduced the viable-cell proportion to approximately 66% and increased dead cells to approximately 34%. The BA + DOX combination produced the greatest change, with approximately 46% viable and 54% dead cells. These findings demonstrate increased cell death following combined BA and DOX treatment compared with either single treatment (Figure 10).

### 3.11. Nuclear Morphological Changes by DAPI Staining

**Figure 11:**
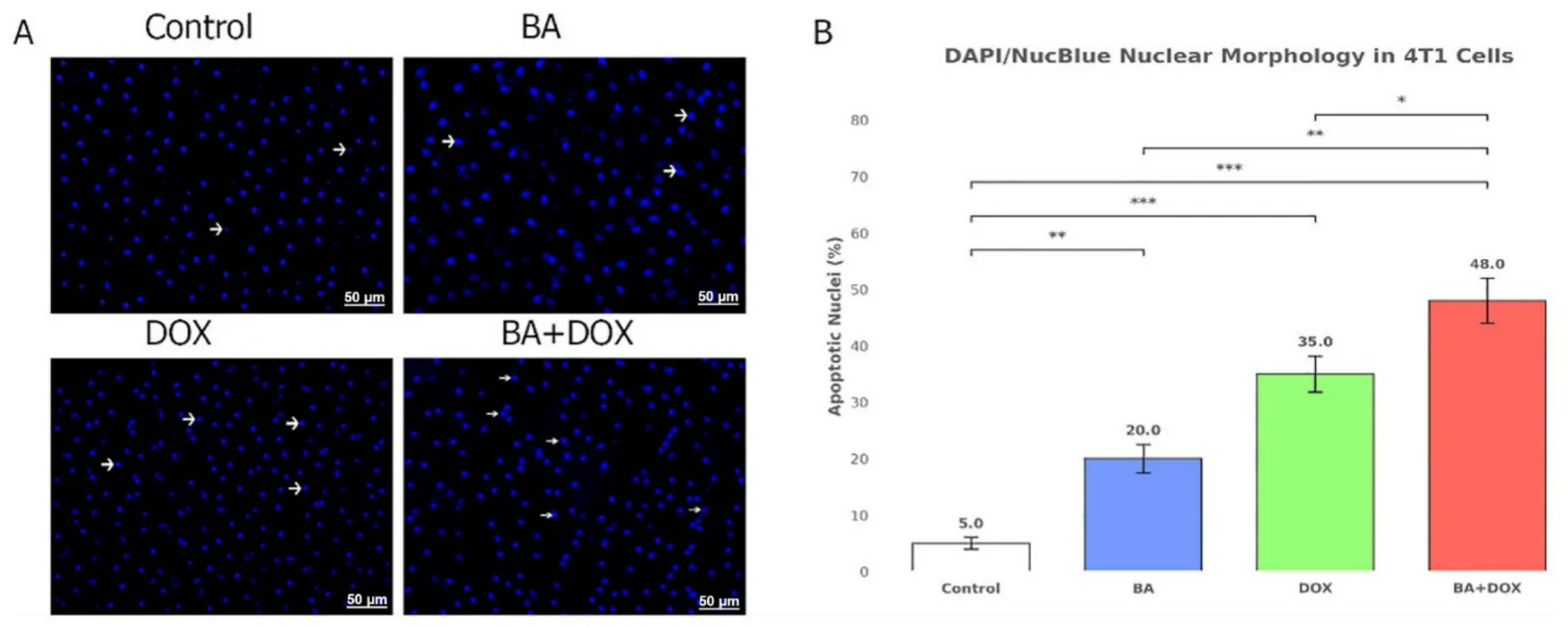
Effect of BA and DOX on nuclear morphology and apoptotic nuclei formation in 4T1 breast cancer cells. (**A**) Representative fluorescence micrographs of DAPI-stained nuclei after 48 h of treatment with BA (30 μM), DOX (1.5 μM), or their combination (BA + DOX; 30 + 1.5 μM). White arrows indicate representative apoptotic nuclei exhibiting chromatin condensation, nuclear shrinkage, and DNA fragmentation. Scale bar = 50 μm. (**B**) Semi-quantitative analysis of apoptotic nuclei expressed as the percentage of total nuclei. Nuclear morphology was evaluated in at least five randomly selected microscopic fields per sample using ImageJ software. Data are presented as mean ± SD from three independent experiments. Statistical significance was determined using one-way ANOVA followed by Tukey’s multiple comparison test. Statistical significance is indicated as *p* < 0.05 (*), *p* < 0.01 (**), and *p* < 0.001 (***).

DAPI staining demonstrated progressive nuclear morphological alterations after BA and DOX treatment in 4T1 cells (Figure 11). The nuclei in the control group were predominantly intact and uniformly stained, with relatively few nuclei displaying apoptotic morphological features. BA treatment increased the frequency of nuclei exhibiting chromatin condensation and nuclear shrinkage, whereas DOX treatment produced more pronounced nuclear condensation and fragmentation. The BA + DOX combination showed the highest frequency of nuclei displaying apoptotic morphological alterations.

Quantitative analysis showed that the percentage of nuclei with apoptosis-associated morphology increased from 5.0 ± 1.4% in the control group to 20.0 ± 3.6% following BA treatment and 35.0 ± 4.2% following DOX treatment, reaching 48.0 ± 4.8% in the BA + DOX group. Thus, the combination treatment was associated with the highest proportion of apoptotic nuclear morphology among the experimental groups (Figure 11).

### 3.12. Findings of the Bioinformatic Analysis

#### 3.12.1. GO Enrichment Analysis

**Figure 12:**
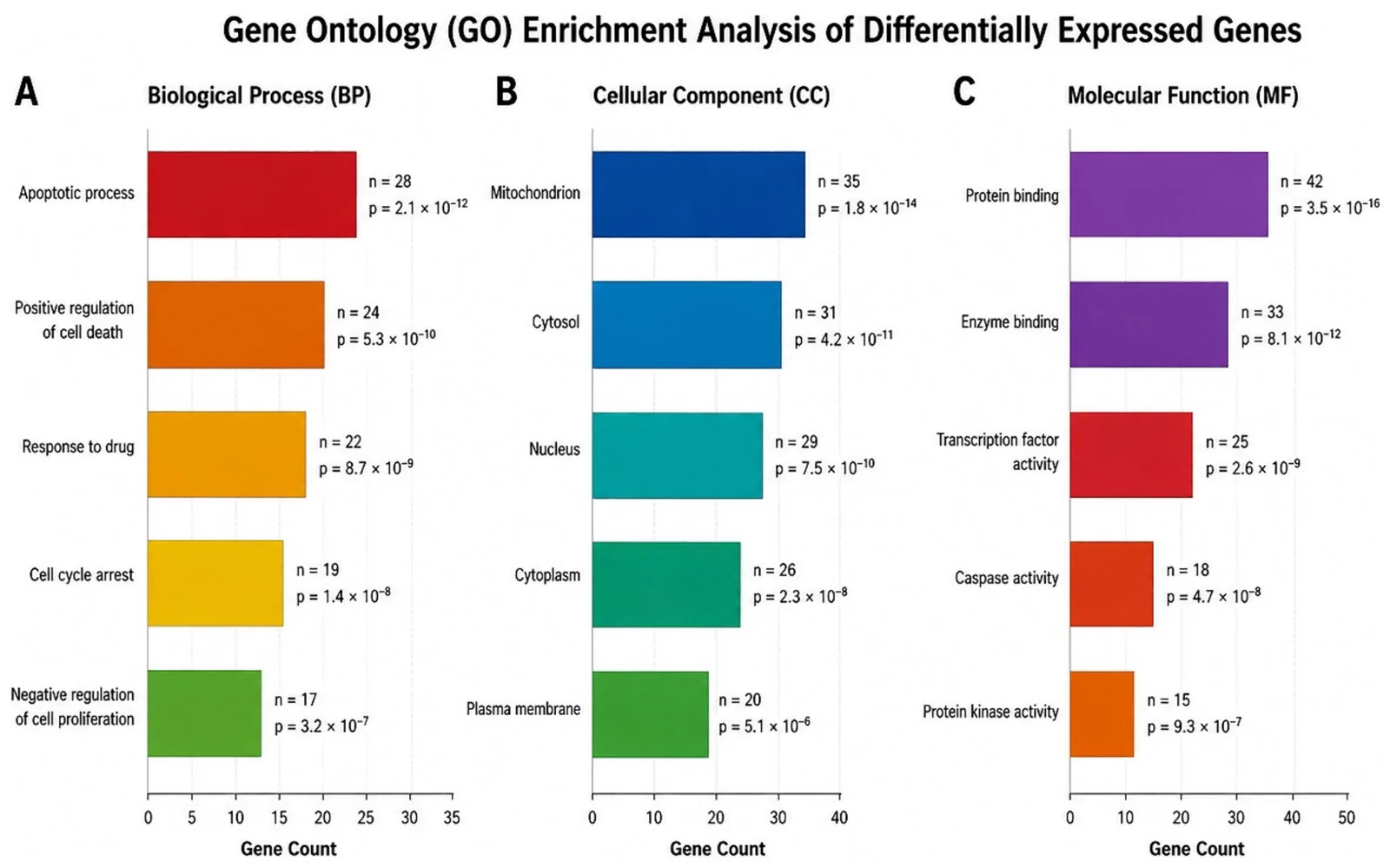
GO enrichment analysis of common target genes predicted to be associated with BA, DOX, and breast cancer. (**A**) BP analysis identified significant enrichment in the apoptotic process, positive regulation of cell death, response to drug, cell cycle arrest, and negative regulation of cell proliferation. (**B**) CC analysis showed enrichment in the mitochondrion, cytosol, nucleus, cytoplasm, and plasma membrane. (**C**) MF analysis identified enrichment in protein binding, enzyme binding, transcription factor activity, caspase activity, and protein kinase activity. The lengths of the bars represent the number of genes associated with each enriched GO term, and the corresponding Benjamini–Hochberg-adjusted FDR values are indicated. Only terms with FDR < 0.05 are shown.

GO enrichment analysis was performed on common target genes predicted to characterise their functional distribution in the BP, CC, and MF categories (Figure 12).

The prominent enriched terms included the apoptotic process (28 genes, FDR = 2.1 × 10^−12^), positive regulation of cell death (24 genes, FDR = 5.3 × 10^−10^), response to drug (22 genes, FDR = 8.7 × 10^−9^), cell cycle arrest (19 genes, FDR = 1.4 × 10 ^−8^) and negative regulation of cell proliferation (17 genes, FDR = 3.2 × 10^−7^). These enrichment patterns indicate that the predicted common targets are associated with biological processes related to apoptosis, cell death, and cell cycle.

In the CC category, enriched terms included mitochondrion (35 genes, FDR = 1.8 × 10^−14^), cytosol (31 genes, FDR = 4.2 × 10^−11^), nucleus (29 genes, FDR = 7.5 × 10^−10^), cytoplasm (26 genes, FDR = 2.3 × 10^−8^), and plasma membrane (20 genes, FDR = 5.1 × 10^−6^). In the MF category, enriched terms included protein binding (42 genes, FDR = 3.5 × 10^−16^), enzyme binding (33 genes, FDR = 8.1 × 10^−12^), transcription factor activity (25 genes, FDR = 2.6 × 10^−9^), caspase activity (18 genes, FDR = 4.7 × 10^−8^), and protein kinase activity (15 genes, FDR = 9.3 × 10^−7^) (Figure 12).

#### 3.12.2. KEGG Pathway Enrichment Analysis

**Figure 13:**
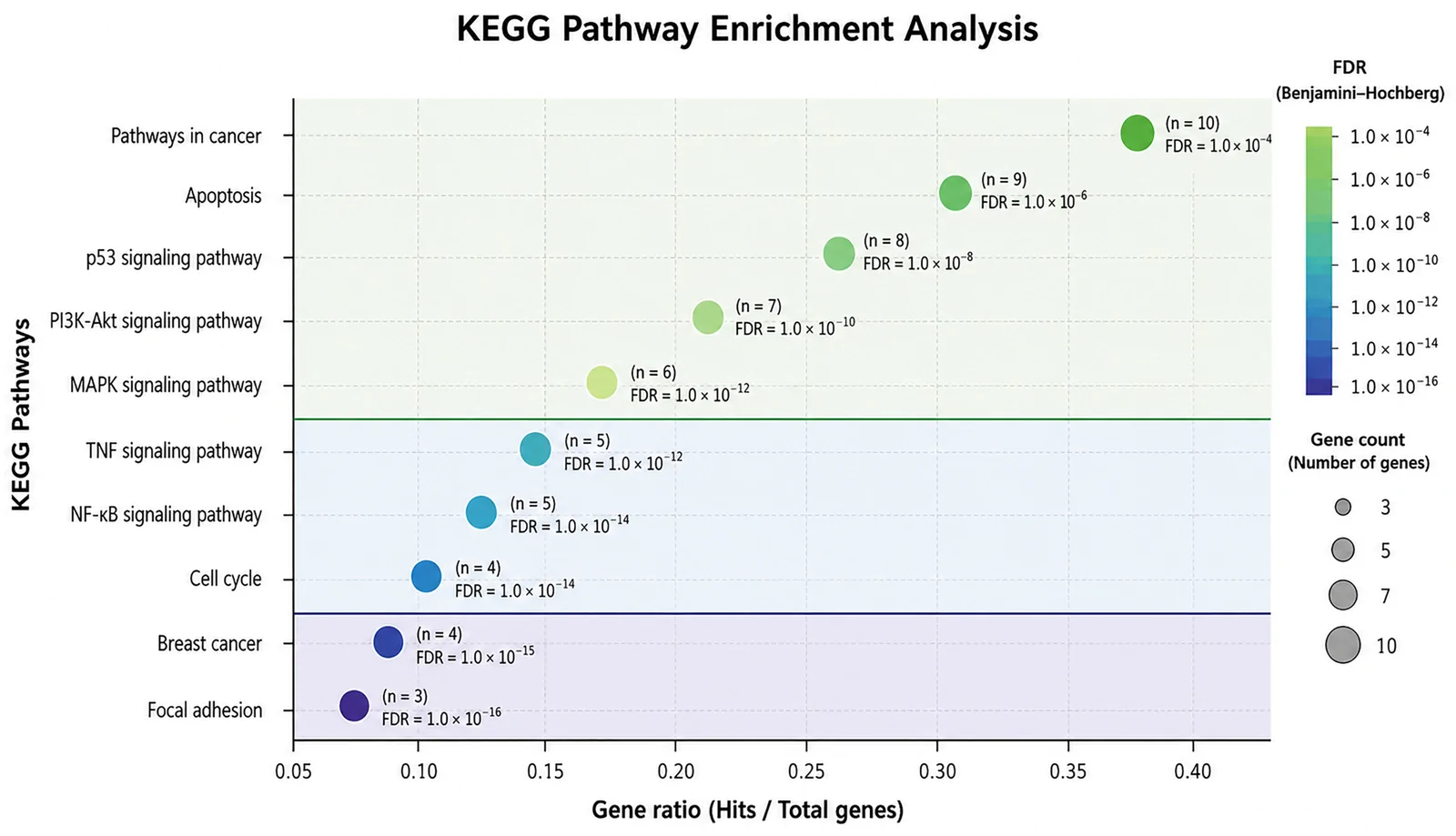
KEGG pathway enrichment analysis of common targets predicted associated with BA, DOX, and breast cancer. The bubble plot shows significantly enriched KEGG pathways identified using the STRING enrichment module. The *x*-axis represents the gene ratio (number of target genes associated with each pathway compared to total analysed genes), the bubble size represents the number of associated genes, and the bubble colour represents the Benjamini–Hochberg-adjusted FDR. The enriched pathways included cancer pathways, apoptosis, p53 signalling pathway, PI3K-Akt signalling pathway, MAPK signalling pathway, TNF signalling pathway, NF-κB signalling pathway, cell cycle, breast cancer, and focal adhesion. Only pathways that meet the predefined significance threshold (FDR < 0.05) are shown.

To explore the biological pathways potentially associated with the predicted common targets of BA, DOX and breast cancer-related genes, the enrichment analysis of the KEGG pathway was performed using the STRING enrichment module (Figure 13).

The enrichment analysis demonstrated that the predicted common targets were significantly enriched in pathways associated with apoptosis, cancer progression, and cell cycle regulation. The pathways most significantly enriched included pathways in cancer, apoptosis, p53 signalling pathway, PI3K–Akt signalling pathway, MAPK signalling pathway, TNF signalling pathway, NF-κB signalling pathway, cell cycle, breast cancer, and focal adhesion (Figure 13).

Among these, the cancer, apoptosis, and p53 signalling pathways exhibited the highest gene ratios and the largest number of enriched genes, together with the lowest FDR values, indicating a strong predicted association with apoptosis-related signalling. The enrichment of the signalling pathways PI3K-Akt, MAPK, TNF, and NF-κB further suggests that these signalling networks may contribute to the regulation of cell survival, proliferation, cell-cycle progression, and apoptosis. Because these findings were derived from an in silico enrichment analysis of predicted common targets rather than experimentally demonstrated BA-dependent molecular changes, they should be interpreted as hypothesis-generating pathway associations rather than evidence of target engagement, pathway activation, or an established molecular mechanism.

#### 3.12.3. PPI Network and Topological Analysis

**Figure 14:**
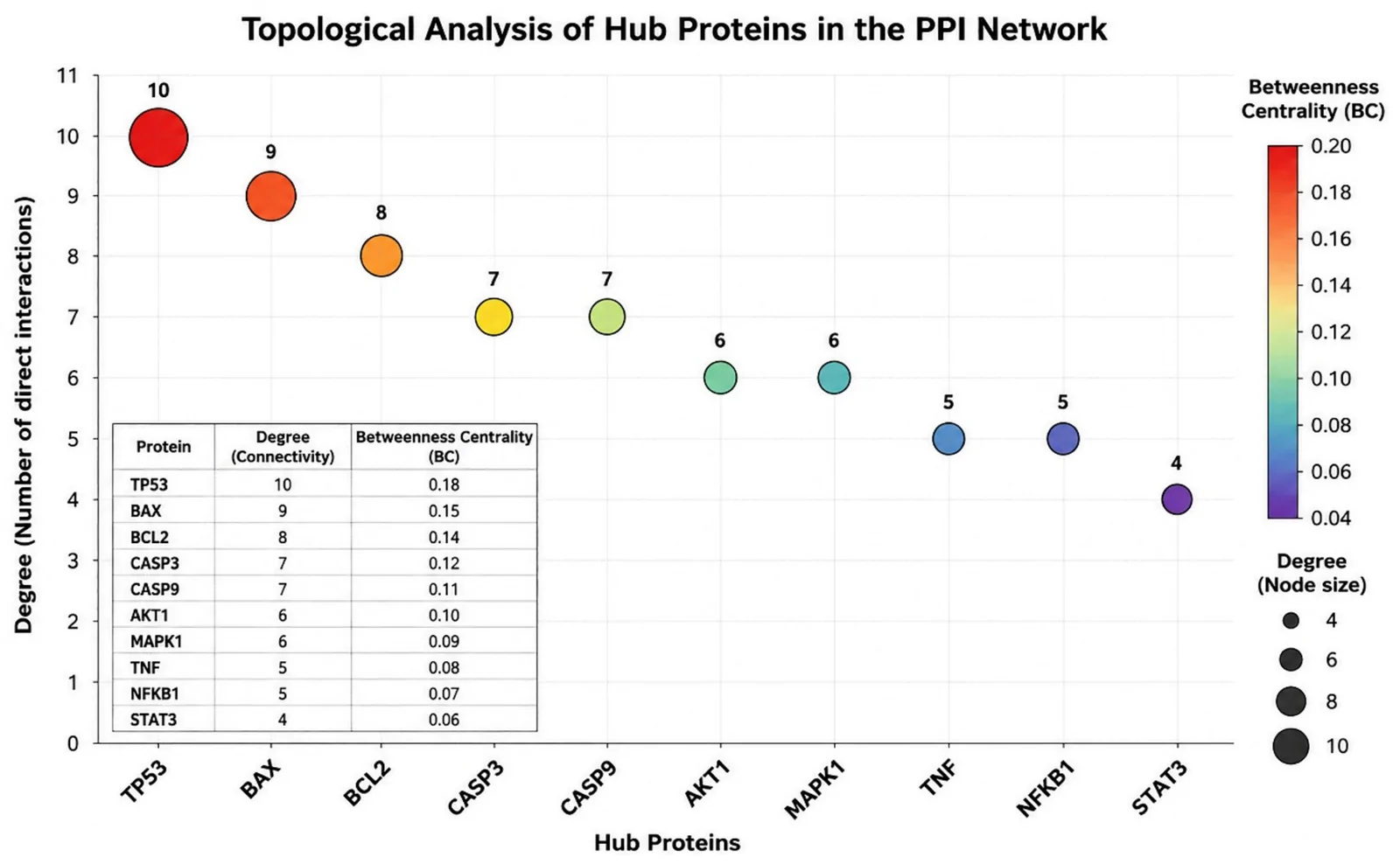
Topological analysis of the top 10 central proteins identified in the PPI network associated with BA and DOX. The hub proteins were classified according to degree (connectivity) and BC. The *x*-axis represents the identified hub proteins, whereas the *y*-axis indicates the degree of the node. The bubble size is proportional to the degree of the node, and the bubble colour represents BC. TP53 showed the highest connectivity (degree = 10; BC = 0.18), followed by BAX (degree = 9; BC = 0.15), BCL2 (degree = 8; BC = 0.14), CASP3 (degree = 7; BC = 0.12), CASP9 (degree = 7; BC = 0.11), AKT1 (degree = 6; BC = 0.10), MAPK1 (degree = 6; BC = 0.09), TNF (degree = 5; BC = 0.08), NFKB1 (degree = 5; BC = 0.07) and STAT3 (degree = 4; BC = 0.06). These topological parameters indicate that TP53, BAX, BCL2, CASP3, and CASP9 were among the proteins most closely connected within the selected PPI network.

PPI network analysis was performed using the STRING database to characterise interactions among selected apoptosis-related hub proteins. The topological analysis identified TP53 as the protein with the highest connectivity, with a degree of 10 and a BC value of 0.18. TP53 was followed by BAX (Degree = 9, BC = 0.15), BCL2 (Degree = 8, BC = 0.14), CASP3 (Degree = 7, BC = 0.12), and CASP9 (Degree = 7, BC = 0.11). AKT1 and MAPK1 each showed a degree of 6, while TNF, NFKB1 and STAT3 exhibited degrees of 5, 5 and 4, respectively (Figure 14).

The prominence of apoptosis-associated proteins, including BAX, BCL2, CASP3, and CASP9, within the predicted interaction network was generally consistent with the experimentally observed changes in apoptosis-related gene expression, caspase-3/7 activity, and mitochondrial membrane potential. However, TP53, AKT1, MAPK1, NFKB1, and STAT3 were not experimentally validated in the present study. Therefore, their positions within the PPI network should be interpreted as predicted molecular associations rather than evidence of pathway activation. Collectively, PPI analysis provides a hypothesis-generating framework linking predicted common targets to apoptosis- and cell survival-related signalling networks.

## 4. Discussion

This study evaluated the cytotoxicity, drug-interaction profile, and treatment-associated cellular responses to BA, DOX, and their combination in the murine 4T1 TNBC-like model, with immortalised HaCaT keratinocytes included as a non-tissue-matched comparative model. Both BA and DOX produced concentration- and time-dependent reductions in cell viability; however, their respective 48 h selectivity indices of 1.28 and 1.56 indicated only modest differential cytotoxicity between 4T1 and HaCaT cells under the experimental conditions. The BA–DOX interaction was concentration- and reference-model-dependent rather than uniformly synergistic across the tested concentration range. Under the selected phenotypic-characterisation condition, which was analytically distinct from the drug-interaction concentration series, combined BA and DOX exposure was associated with the highest measured apoptotic fraction, increased Sub-G1 and G2/M fractions, mitochondrial membrane depolarisation, increased caspase-3/7 activity, increased expression of the pro-apoptotic genes *Bax*, *Casp3*, and *Casp9*, decreased expression of *Bcl2*, and an increased *Bax*/*Bcl2* mRNA ratio. Calcein-AM/PI and DAPI analyses additionally demonstrated increased cell death and apoptosis-associated nuclear alterations after combined treatment. The measured concentrations of TNF-α and IL-6 were lower following BA + DOX exposure, whereas IL-10 remained unchanged. These cellular and molecular endpoints characterise a downstream apoptosis-associated phenotype but do not identify a direct molecular target of BA or establish a causal mitochondrial apoptotic mechanism. Similarly, the GO, KEGG, and PPI findings represent predicted pathway and network associations and cannot substitute for experimental target identification or functional pathway validation. Accordingly, the contribution of the present study lies in the quantitative characterisation of the concentration- and model-dependent BA–DOX interaction profile and the cellular phenotype accompanying the selected combined-exposure condition, rather than in the discovery of a previously unknown anticancer property or direct molecular mechanism of BA. The findings also do not establish TNBC-specific activity or definitive cancer-cell selectivity.

The BA–DOX drug-interaction profile varied according to concentration and analytical model. According to the predefined operational CI criteria, the lowest concentration pair (5 + 5 µM; CI = 1.140) was classified as near-additive, whereas the 10 + 10 µM pair (CI = 1.276) was classified as antagonistic. At the intermediate and higher concentration pairs, CI values below 0.8 were classified as synergistic. Nevertheless, the HSA and Bliss models produced divergent results, demonstrating that the BA–DOX combination cannot be characterised as uniformly synergistic across the tested concentration range or independently of the selected reference model. Importantly, the 30 µM BA plus 1.5 µM DOX condition used for subsequent phenotypic characterisation was selected on the basis of the individual 48 h dose–response profiles and approximate single-agent IC_50_ values. This concentration pair was not included in the fixed-ratio CI analysis and should therefore not be interpreted as a pharmacologically validated synergistic condition or as evidence of DOX chemosensitisation. Under this selected condition, combined exposure was associated with greater measured apoptotic and downstream cellular responses than either single treatment, including increased Annexin V-positive populations, mitochondrial membrane depolarisation, increased caspase-3/7 activity, increased expression of *Bax*, *Casp3*, and *Casp9*, decreased expression of *Bcl2*, and an increased *Bax*/*Bcl2* mRNA ratio. Previous investigations in the 4T1 model have reported cytotoxic and apoptosis-associated responses to natural bioactive compounds and DOX-based interventions, providing model-specific biological context for the present observations but not direct evidence of a BA–DOX interaction [19,20,21]. Toprak et al. similarly reported that thymoquinone combined with DOX reduced cell viability and shifted apoptosis-related gene expression toward a pro-apoptotic profile in OVCAR-3 cells [22]. However, differences in compound identity, cellular model, treatment concentrations, and experimental design preclude direct mechanistic or pharmacological extrapolation. Thus, these comparisons support the biological plausibility of examining natural compound–chemotherapy combinations but do not establish a direct target-level mechanism or a general chemosensitising effect of BA.

Duman et al. reported that quercetin combined with mitomycin C in retinoblastoma cells produced CI values below 1, together with G2/M accumulation, increased apoptosis, upregulation of *BAX*, *TP53*, and *CASP3*, downregulation of *BCL2*, and reduced VEGF and IL-6 secretion [23]. The direction of some of these treatment-associated changes is broadly comparable to the increased *Bax*/*Bcl2* mRNA ratio and lower measured cytokine concentrations observed in the present study. However, these similarities involve different natural compounds, chemotherapeutic agents, and cancer models and therefore do not demonstrate a shared molecular mechanism or a BA-specific biological property. Instead, they provide contextual evidence that natural compound–chemotherapy combinations can be accompanied by multiple downstream cellular responses in addition to changes in cell viability.

In a study conducted by the same research group and directly related to BA, BA combined with gemcitabine (GEM) was evaluated in human endometrial cancer (ECC-1) cells under hypoxic and normoxic conditions. BA + GEM produced synergistic cytotoxicity under both conditions, suppressed HIF-1α and VEGF expression, increased *Bax* and *Caspase-3* expression while decreasing *Bcl-2* expression, and increased caspase-3/7, -8, and -9 activities [12]. These previous findings provide biological context for investigating BA-containing combinations but do not identify the molecular target responsible for the effects of BA or establish that the same responses occur in the 4T1 model. The BA concentration range used in the present study (5–50 µM) also produced a clear dose–response pattern, broadly consistent with the IC_50_ range reported in that study (~35–42 µM). Furthermore, the GO enrichment reported in that study and the apoptosis-related GO/KEGG associations observed here provide only comparative, hypothesis-generating context and do not establish a common BA-dependent mechanism. In another study by the same research team, curcumin was shown to exert inhibitory effects in cervical cancer cells in association with the RAS/RAF signalling pathway [7]. However, because this finding involved a different natural compound and cancer model, it cannot be used to infer a BA-specific signalling mechanism in 4T1 cells. Comprehensive reviews of the anticancer activities of boswellic acids have similarly described effects on cell-cycle regulation, apoptosis, and signalling networks involving p53, Akt, and NF-κB [10,13]. These observations provide a biological context for the dose-dependent cytotoxicity of BA observed in 4T1 cells, but do not establish direct modulation of these pathways in the present model. The mitochondrial membrane depolarisation, increased caspase-3/7 activity, altered *Bax*/*Bcl2* mRNA ratio, and increased *Casp9* and *Casp3* expression observed in the present study should therefore be interpreted as components of a treatment-associated apoptotic phenotype rather than as evidence identifying a direct molecular target or establishing a causal mitochondrial mechanism. Similar changes in apoptosis-related markers have also been reported in combination studies containing BA [12].

Regarding cytokine measurements, the concentrations of TNF-α and IL-6 measured in the culture supernatants were lower following BA and DOX treatment, with the lowest concentrations observed after combined treatment, while IL-10 remained unchanged. Changes in inflammatory cytokines have also been reported in the BA + GEM study, including reductions in IL-1β, IL-6, and TNF-α [12]. However, because the present experiments were carried out in 4T1 monocultures without immune or stromal components, and because the cytokine measurements were not normalised to viable cell number or total cellular protein, these findings should be interpreted as differences in bulk supernatant cytokine concentrations rather than as evidence of specific regulation of cytokine secretion. Treatment-related loss of viable cells may have contributed to the lower measured TNF-α and IL-6 concentrations. Accordingly, these results do not demonstrate modulation of the tumour microenvironment or identify an anti-inflammatory mechanism of BA.

In terms of the non-malignant cell comparison, the comparatively lower cytotoxicity observed in HaCaT cells corresponded to a 48 h SI of only 1.28 for BA and therefore indicates modest differential cytotoxicity under the present experimental conditions rather than definitive cancer-cell selectivity. Because HaCaT cells are immortalised keratinocytes and are not tissue-matched normal mammary epithelial cells, this comparison cannot establish the safety of BA toward normal breast tissue or demonstrate that BA reduces DOX-associated systemic toxicity. Consequently, the findings should be considered preliminary and require validation using appropriate normal mammary epithelial models and in vivo systems.

In the mitochondrial apoptotic pathway, the BCL-2 family proteins are key regulators of the cellular balance between survival and death. Increased pro-apoptotic BAX relative to anti-apoptotic BCL-2 favours mitochondrial outer membrane permeabilization, which can promote the release of cytochrome *c* and subsequent activation of CASP9 and executioner caspases. Current reviews indicate that the Bax/Bcl-2 balance is an important marker of treatment-associated apoptotic responses in breast cancer and that natural compounds can shift this balance to a pro-apoptotic state [23,24,25]. However, the present study quantified *Bax* and *Bcl2* at the mRNA level, and the resulting transcript ratio cannot be assumed to represent the abundance, localisation, or functional activity of the corresponding BAX and BCL-2 proteins. In the present study, BA alone altered apoptosis-related mRNA expression, while the greatest changes were observed after combined BA and DOX treatment. DOX is well established as a DNA-damaging chemotherapeutic agent capable of inducing apoptotic signalling, while natural bioactive compounds have been reported to influence oxidative stress and PI3K/AKT, MAPK and p53-associated signalling networks. Because these signalling networks were not experimentally assessed in the present study, their modulation by BA or BA + DOX cannot be inferred from the observed transcriptional changes. Previous studies have also shown that combinations of natural products and chemotherapy can produce greater apoptotic responses than either treatment alone [26].

Recent studies investigating the apoptotic effects of natural products in breast cancer have emphasised that treatment responses are better characterised using multiple complementary apoptotic endpoints rather than a single molecular marker. In particular, phytochemicals have been reported to increase BAX expression, suppress BCL2 expression, and enhance apoptotic signalling associated with CASP3/CASP9 [27]. The RT-qPCR findings of the present study are broadly consistent with this pattern. Caspase-3 activation has also been associated with responses to chemotherapeutic agents in breast cancer [24,28]. In our study, BA increased caspase-3/7 activity, DOX produced a greater increase, and the highest activity was observed in the BA + DOX group. Importantly, this functional caspase-3/7 assay supports increased executioner caspase activity, but does not constitute direct protein-expression validation of CASP3 or CASP7. Consequently, the concordance between the RT-qPCR and caspase-3/7 findings should be interpreted as complementary evidence of increased apoptotic signalling rather than confirmation of transcriptional changes at the protein level. Furthermore, increased caspase-3/7 activity represents a downstream effector response and does not identify the upstream molecular target of BA or establish the causal pathway responsible for the greater response observed after combined exposure.

Combinations of natural triterpenoids with chemotherapeutic agents have also been associated with caspase-dependent apoptosis. Betulinic acid, for example, has been reported to increase mitochondrial membrane permeability, promote cytochrome *c* release, and subsequently activate CASP9 and CASP3. Natural compounds have also been reported to enhance caspase activation and modify chemotherapy responses through mechanisms associated with PI3K/AKT [26,28]. However, betulinic acid is distinct from β-boswellic acid, and the PI3K/AKT pathway was not experimentally evaluated in the present study; therefore, these previous findings provide only general biological context and cannot be considered evidence of a BA-specific mechanism in 4T1 cells.

The findings obtained using the different experimental approaches in the present study were internally consistent. The increased populations of apoptotic cells detected by Annexin V-FITC/PI staining were accompanied by mitochondrial membrane depolarisation demonstrated by JC-1 analysis, increased caspase-3/7 activity, increased mRNA expression of *Bax*, *Casp3* and *Casp9*, decreased mRNA expression of *Bcl2*, and an increased *Bax*/*Bcl2* mRNA ratio. DAPI staining further demonstrated nuclear alterations associated with apoptosis, including chromatin condensation and nuclear fragmentation. Collectively, these complementary endpoints describe an apoptosis-associated cellular phenotype accompanied by mitochondrial membrane depolarisation following combined BA and DOX exposure [29]. However, JC-1 analysis demonstrates a change in mitochondrial membrane potential and does not, by itself, confirm activation of the intrinsic mitochondrial apoptotic pathway. In the absence of direct assessment of cytochrome *c* release, apoptosis-related protein abundance or cleavage, and functional pathway inhibition or rescue, these findings cannot establish a causal mitochondrial mechanism or identify the direct molecular target of BA. Previous studies investigating natural triterpenoids have described chromatin condensation and other apoptotic nuclear alterations. Betulinic acid has been reported to alter mitochondrial function, promote cytochrome *c* release, and induce caspase-associated DNA fragmentation [30]. Because betulinic acid and β-boswellic acid are distinct compounds, this comparison provides contextual support for the observed cellular phenotype but not direct mechanistic evidence for BA. Similarly, combinations of natural products with DOX have been associated with more pronounced chromatin condensation and apoptotic body formation than single treatments [25]. The morphological findings obtained in the present study are broadly consistent with these observations.

The enrichment of mitochondria-associated terms in the GO analysis was also noteworthy. Mitochondria are central regulators of intrinsic apoptotic signalling, and increased mitochondrial outer membrane permeability can initiate cytochrome *c* release followed by the activation of apoptotic caspases [31,32]. In the present study, mitochondrial membrane depolarisation detected by JC-1 analysis, together with increased mRNA expression of *Bax*, *Casp9*, and *Casp3* and increased caspase-3/7 activity, showed qualitative correspondence with the mitochondrial enrichment identified by GO analysis. However, the GO analysis was based on database-predicted common targets rather than experimentally measured BA-dependent molecular changes. It therefore neither validates the experimental findings nor constitutes direct mechanistic evidence. The PI3K/AKT and MAPK signalling pathways identified by KEGG analysis also represent potential molecular networks of interest. These pathways regulate proliferation, survival, cellular stress responses, and apoptosis, and natural triterpenoids have been reported to influence PI3K/AKT and MAPK-associated signalling [27,33]. However, in the present study, neither pathway was experimentally assessed; therefore, the KEGG findings should be considered hypothesis-generating rather than evidence of pathway activation or inhibition or identification of a BA-specific molecular target.

The PPI analysis provided an additional exploratory framework for interpreting the predicted common targets. The highly connected proteins within the selected network included TP53, BAX, BCL2, CASP3, CASP9, AKT1, MAPK1, TNF, NFKB1, and STAT3 [34]. The prominence of BAX, BCL2, CASP3, and CASP9 within the predicted network showed qualitative overlap with the experimentally observed changes in the mRNA expression of genes related to apoptosis and caspase-3/7 activity. However, this overlap should not be interpreted as independent experimental confirmation because the network was constructed from predicted targets and previously documented protein associations rather than BA-dependent molecular changes measured in 4T1 cells. STRING interactions integrate information from the literature, experimental databases, co-expression, and computational predictions and do not directly represent protein interactions occurring specifically in 4T1 cells. Moreover, TP53, AKT1, MAPK1, NFKB1, and STAT3 were not experimentally validated in the present study. Therefore, their network positions should be interpreted as predicted molecular associations that may guide future validation studies, rather than as identified BA targets or direct evidence of pathway activation.

This study has several limitations that should be acknowledged. First, all experiments were performed in vitro using a single murine breast cancer cell line (4T1). Therefore, the findings may not fully represent the biological heterogeneity of breast cancer or the complexity of the tumour microenvironment in vivo. In addition, HaCaT keratinocytes were used as a non-malignant control; however, they do not represent a matched normal mammary epithelial cell model, and therefore conclusions regarding tumour selectivity should be interpreted with caution. Second, phenotypic characterisation was performed using only a single treatment condition (30 µM BA + 1.5 µM DOX) and a single 48 h time point, precluding assessment of dose- and time-dependent cellular and molecular responses. Third, the present study did not identify a direct molecular target of BA or establish a causal signalling pathway linking BA exposure to the greater response observed after combined treatment. Although apoptosis-related gene expression, mitochondrial membrane depolarisation, caspase-3/7 activity, nuclear morphology, and apoptosis were evaluated, these measurements represent downstream treatment-associated endpoints. Key signalling pathways were not validated at the protein level by Western blotting or related assays (e.g., cytochrome *c* release, PARP cleavage, Apaf-1 expression, or pathway inhibition studies). Direct target-engagement analyses and pharmacological or genetic perturbation and rescue experiments were also not performed. Consequently, the findings describe an apoptosis-associated cellular phenotype but do not establish the direct mechanism of action of BA. Fourth, cytokine concentrations were not normalised to viable cell number, total cellular protein, or another measure of cell abundance. Therefore, treatment-related loss of viable cells may have contributed to the lower TNF-α and IL-6 concentrations measured in the culture supernatants. Fifth, all quantitative experiments were based on three independent biological replicates. Although technical replicates were averaged within each biological experiment, the limited biological sample size restricts the precision and generalisability of the statistical estimates, and the findings should therefore be considered exploratory. Sixth, the interaction between BA and DOX was concentration dependent. Although the Chou–Talalay model demonstrated synergism in selected concentration pairs, antagonism was observed at lower concentrations, and the phenotypic-characterisation condition (30 µM BA + 1.5 µM DOX) was not directly evaluated in the CI analysis. Furthermore, different drug-interaction models (Chou–Talalay, HSA and Bliss) yielded partially divergent results, highlighting the importance of interpreting synergistic interactions with caution.

## 5. Conclusions

In conclusion, combined BA and DOX exposure produced greater measured cytotoxic and apoptosis-associated responses than either single treatment in 4T1 TNBC-like cells under the selected 48 h phenotypic-characterisation condition. However, the BA–DOX interaction was concentration- and analytical-model-dependent, and the 30 µM BA plus 1.5 µM DOX condition was not included in the CI analysis. Therefore, the greater response observed under this condition should not be interpreted as pharmacologically validated synergy, chemosensitisation, or evidence of a DOX dose-sparing effect. The combined treatment reduced cell viability, increased apoptotic cell death, was accompanied by mitochondrial membrane depolarisation, increased caspase-3/7 activity, increased the mRNA expression of *Bax*, *Casp3*, and *Casp9*, decreased *Bcl2* mRNA expression, and markedly increased the *Bax*/*Bcl2* mRNA ratio. These findings were further supported by increased PI-positive cell death and nuclear alterations associated with apoptosis. Bioinformatic analyses identified predicted enrichment of apoptosis-, mitochondrial- and cell cycle-related processes and signalling pathways, providing hypothesis-generating molecular context for the experimental findings. However, these database-derived associations do not demonstrate BA-dependent target engagement or pathway activation. Collectively, the experimental findings describe a downstream apoptosis-associated cellular phenotype accompanied by mitochondrial membrane depolarisation but do not identify a direct molecular target of BA, establish a causal mitochondrial apoptotic mechanism, or demonstrate TNBC-specific selectivity. Validation in additional breast cancer models, tissue-matched non-malignant mammary cells, and in vivo systems, together with protein-level validation, direct target-engagement analyses, and functional pathway perturbation and rescue experiments, is required before the mechanism and translational relevance of the combination can be established.

## Figures and Tables

**Table 1:** Primer sequences used for qRT-PCR analysis.

Gene	Forward Primer (5′ → 3′)	Reverse Primer (5′ → 3′)
*Bcl2*	ATGCCTTTGTGGAACTATATGGC	GGTATGCACCCAGAGTGATGC
*Bax*	CCCGAGAGGTCTTTTTCCGAG	CCAGCCCATGATGGTTCTGAT
*Casp9*	GAAGCGAATCAATGGACTCGG	CTTGCACTCCTGCATCAGCTT
*Casp3*	AGACAGCCACTCACCTCTTCAG	TTCTGCCAGTGCCTCTTTGCTG
*Gapdh*	GTCTCCTCTGACTTCAACAGCG	ACCACCCTGTTGCTGTAGCCAA

## Data Availability

The original contributions presented in this study are included in the article. Further inquiries can be directed to the corresponding author.

## Abbreviations

ANOVA	Analysis of variance
BA	β-Boswellic acid
BC	Betweenness centrality
BP	Biological Process
CC	Cellular Component
CI	Combination Index
DAPI	4′,6-Diamidino-2-phenylindole
DMSO	Dimethyl sulfoxide
DOX	Doxorubicin
Fa	Fraction affected
FDR	False discovery rate
FITC	Fluorescein isothiocyanate
GAPDH	Glyceraldehyde-3-phosphate dehydrogenase
GO	Gene Ontology
HSA	Highest Single Agent
IC_50_	Half-maximal inhibitory concentration
KEGG	Kyoto Encyclopedia of Genes and Genomes
MF	Molecular Function
OMIM	Online Mendelian Inheritance in Man
PI	Propidium iodide
PPI	Protein–protein interaction
ROS	Reactive oxygen species
SI	Selectivity index
TNBC	Triple-negative breast cancer
